# Supplementary material for: Post‐transcriptional polyadenylation site cleavage maintains 3′‐end processing upon DNA damage
Source: EMBO J. 2023 Feb 10;42(7):e112358. doi: 10.15252/embj.2022112358 (PMC10068322; doi:10.15252/embj.2022112358)
Supplement: Supplementary file 1 — Appendix [file EMBJ-42-e112358-s001.pdf]

APPENDIX

Uncoupling from transcription protects polyadenylation site cleavage from inhibition by DNA damage

Rym Sfaxi[1,2,3,#], Biswendu Biswas[1,2,3,4,5,#], Galina Boldina[1,2,3], Mandy Cadix[1,2,3], Nicolas Servant[6], Huimin Chen[7], Daniel R. Larson[7], Martin Dutertre[1,2,3], Caroline Robert[4,5], Stéphan Vagner[1,2,3,*]

[1]Institut Curie, PSL Research University, CNRS UMR3348, INSERM U1278, F-91405, Orsay, France ;
[2]Université Paris Sud, Université Paris-Saclay, CNRS UMR3348, INSERM U1278, F-91405 Orsay, France ;
[3]Equipe Labellisée Ligue Contre le Cancer;
[4]INSERM U981, Gustave Roussy, Villejuif, France;
[5]Université Paris Sud, Université Paris-Saclay, Kremlin-Bicêtre, France.
[6]INSERM U900, Institut Curie, PSL Research University, Mines ParisTech, 26 rue d'Ulm, 75005, Paris, France ;
[7]Laboratory of Receptor Biology and Gene Expression, National Cancer Institute, NIH, Bethesda, MD 20892, USA

[#]Joint first authors
[*]Corresponding author: Stephan.Vagner@curie.fr

Table of contents

Appendix Figure S1. ***siRNA-mediated depletion of CstF64, CFIm25 and CPSF160 inhibits p53 PAS cleavage in response to UV***......3

Appendix Figure S2. ***Distribution of spots per cell in the smFISH experiments***......4

Appendix Figure S3. ***Depletion of DHX36 or hnRNP H/F inhibits p53 PAS cleavage in the nucleoplasm***......5

Appendix Figure S4. ***CRISPR sgRNAs were used to delete the p53 CoTC element***......6

Appendix Figure S5. ***Partial deletion of the CoTC element inhibits p53 PAS cleavage in response to UV***......7

Appendix Figure S6. ***Partial deletion of the CoTC element inhibits the nucleoplasmic processing of p53 in response to UV***......9

Appendix Figure S7. ***Partial deletion of the CoTC element inhibits the expression of p53 as well as p21 in response to UV***......11

Appendix Figure S8. ***Partial deletion of the CoTC element inhibits cell cycle progression in response to UV.***......12

Appendix Figure S9. ***CoTC elements were identified in the 3' flanking regions of candidate genes***......13

Appendix Figure S10. ***Partial deletion of the CoTC element inhibits p53 PAS cleavage in response to doxorubicin***......16

Appendix Figure S11. ***Validation of PAS cleavage inhibition of candidate pre-mRNAs in response to doxorubicin***......17

Appendix Table S1. ***siRNA sequences for all genes tested***......18

Appendix Table S2. ***20-mer protospacer sequences for two sgRNA and their reverse complement for the deletion of p53 CoTC***......19

Appendix Table S3. ***Modified sgRNA sequences to facilitate cloning***......20

Appendix Table S4. ***Primer sequences used for all genes tested***......21

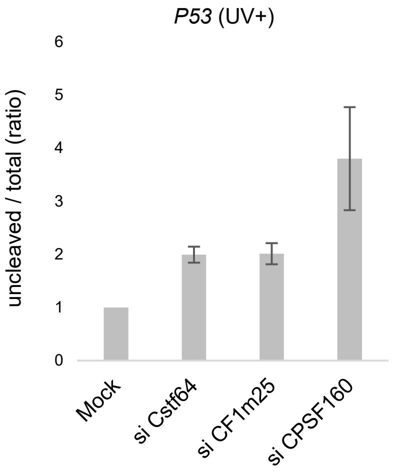

Appendix Figure S1. ***siRNA-mediated depletion of CstF64, CFIm25 and CPSF160 inhibits p53 PAS cleavage in response to UV.*** RT-qPCR assay on nuclear RNA for assessing the uncleaved/total ratio of *p53* pre-mRNA in A549 cells transfected for 48 hours with siRNAs targeting the Cstf64, CF1m25 and CPSF160 and exposed to UV irradiation (40 J/m2) (n=3 technical replicates).

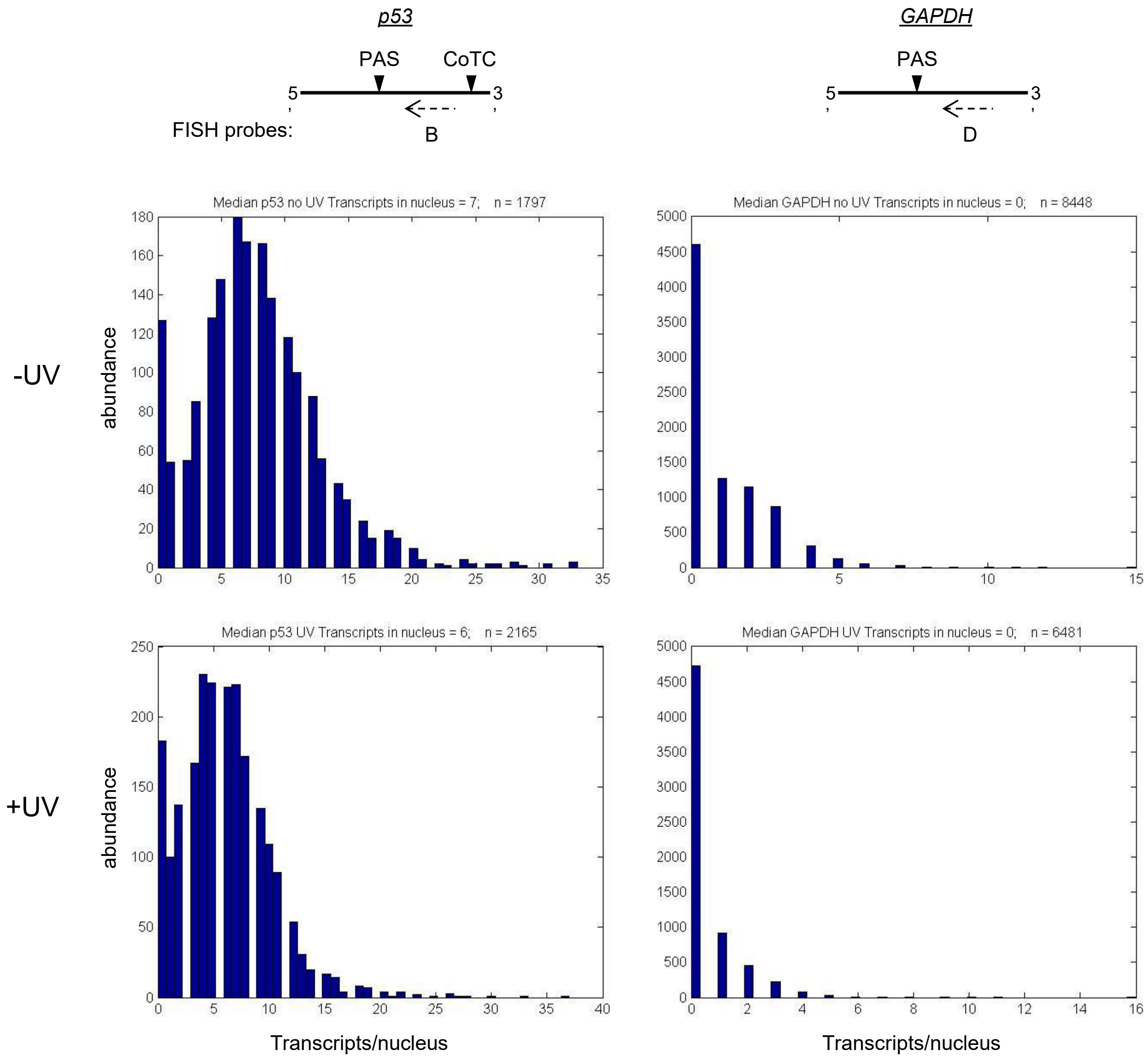

Appendix Figure S2. ***Distribution of spots per cell in the smFISH experiments.*** Distribution of spots per cell in the smFISH experiments (related to Figure 3).

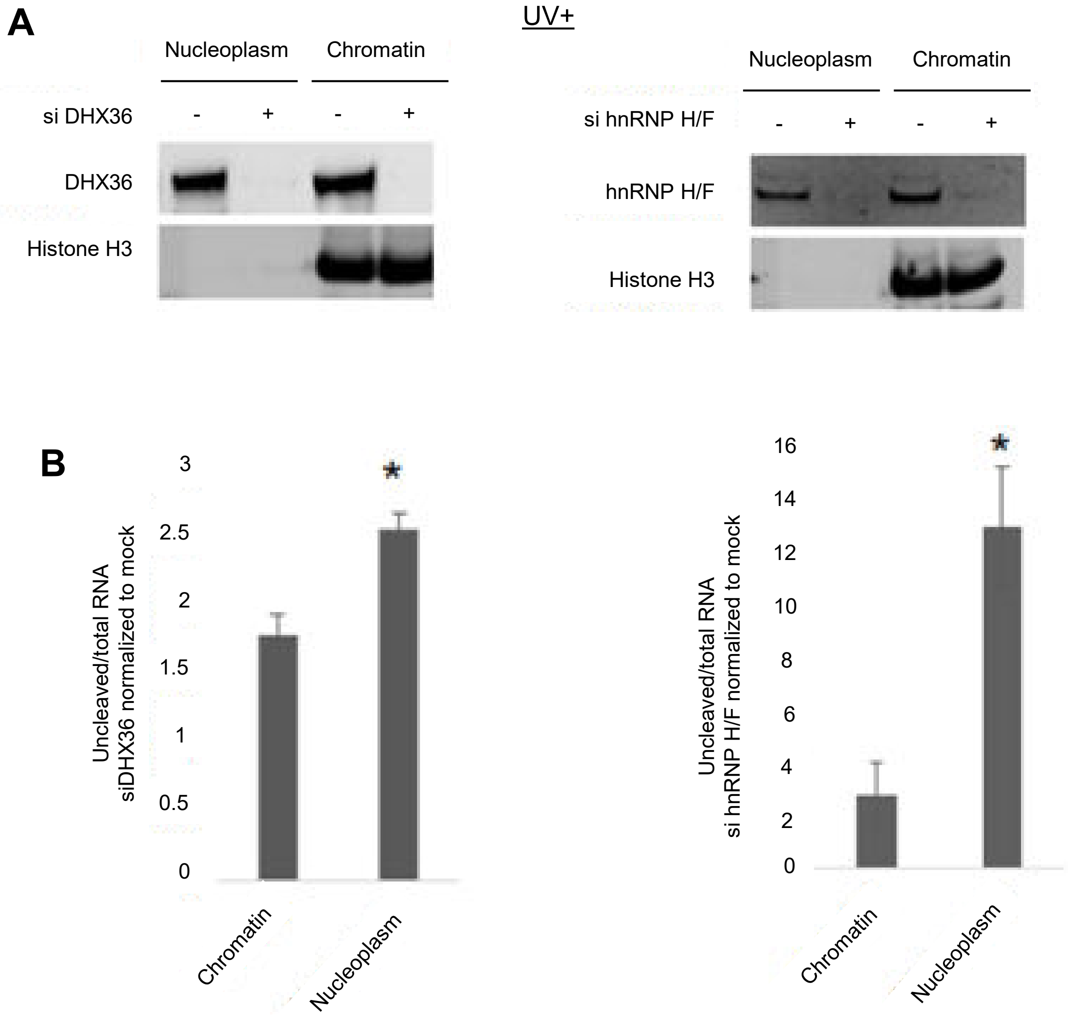

Appendix Figure S3. ***Depletion of DHX36 or hnRNP H/F inhibits p53 PAS cleavage in the nucleoplasm.*** A549 cells were transfected with siRNA against hnRNP H/F 48 hours prior to UV irradiation (40 J/m2). The nucleus was fractionated into nucleoplasm and chromatin following 16 hours of recovery. (A) The depletion efficiency was verified by western blot. (B) RT-qPCR on RNA extracted from the nucleoplasm and the chromatin fraction to quantify the uncleaved/total ratio in both fractions. (n=3 biological replicates) All data are presented as the mean ± s.e.m. *P<0.05

A

Deletion screening primer (forward) sgRNA A

5’ GTCCCTA***CCCAGCAGGCAAACTAGAG***CTCCTGAAGCTCAGTCC**CTGTCCTTGCCTCTGTAGAC**AGGTCACCTTGA 3’

3’ CAGGGATGGGTCGTCCGTTTGATCTCGAGGACTTCGAGTCAGGGACAGGAACGGAGACATCTGTCCAGTGGAACT 5’

p53 CoTC element

5’ TGA**GCTTCCTTTTTTTTTTTTTTAATTTTTTTTTATTTTAGGCTTTATT**GGGGCATAATTGATCCCCCAAAATTGCATACA 3’

3’ ACTCGAAGGAAAAAAAAAAAAAATTAAAAAAAAATAAAATCCGAAATAACCCCGTATAACTAGGGGGTTTTAACGTATGT 5’

5’ TTCAAGGTATGCAGTGTGATGATTTGATATGGGGGTATATTGTGAAACCATTACCACAATCAAATTAATCAGCACGTCC 3’

3’ AAGTTCCATACGTCACACTACTAAACTATACCCCCATATAACACTTTGGTAATGGT**GTTAGTTTAATTAGTCGTGC**AGG 5’

sgRNA B

5’ ATCATCACACACAGTTACCATTTGTGTGTGTGCACGTGTGTTCACCTACGACGAGGACACTTGGACCTACTCTGCAGAT 3’

3’ TAGTAGTGTGTGTCAAGGTAAACACACACACGTGCACACAAGTGGATGCTGCTCCTGTGAACCTGGATGAGACGTCTA 5’

5’ CTCAAGTAAACAGAAAATCTCCCTTTTTGACAACCATCCTCCACCCTTTCAATCCC 3’

3’ GAGTTCATTTGTCTTTTAGAGGGAA***AAACTGTTGGTAGGAGGTGG***GAAAGTTAGGG 5’

Deletion screening primer (reverse)

B

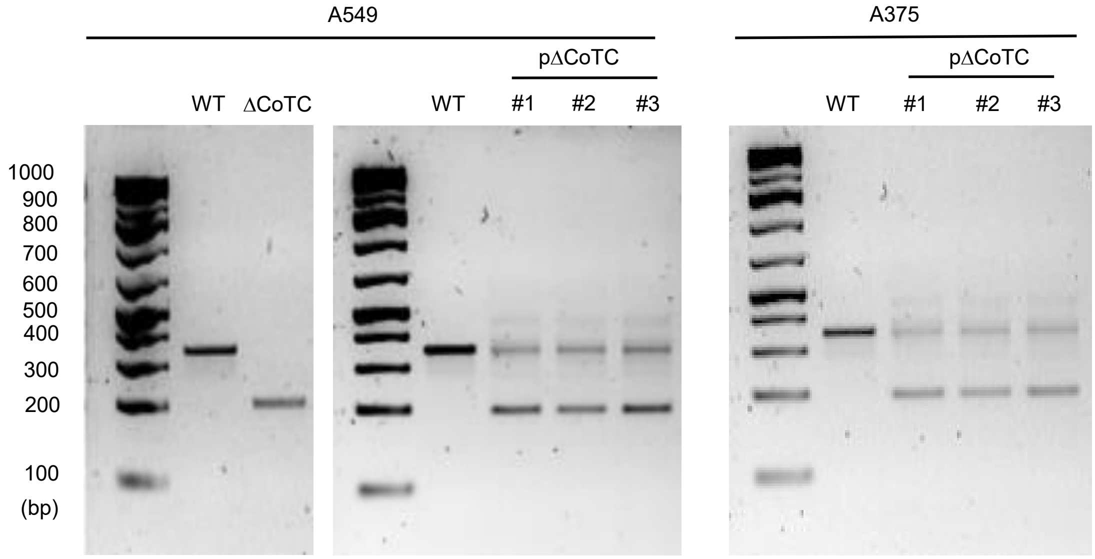

Appendix Figure S4. ***CRISPR sgRNAs were used to delete the p53 CoTC element***

A. CRISPR sgRNAs designed for the CoTC element deletion of the p53 gene.

B. PCR band profile for ‘deletion’ bands for gDNA from wild type (WT) and CRISPR transfected cells. The profile shows bands for complete deletion (ΔCoTC) of the p53 CoTC element in A549 cells as opposed its partial deletion (pΔCoTC) in A549 and A375 cells. (n=3 biological replicates)

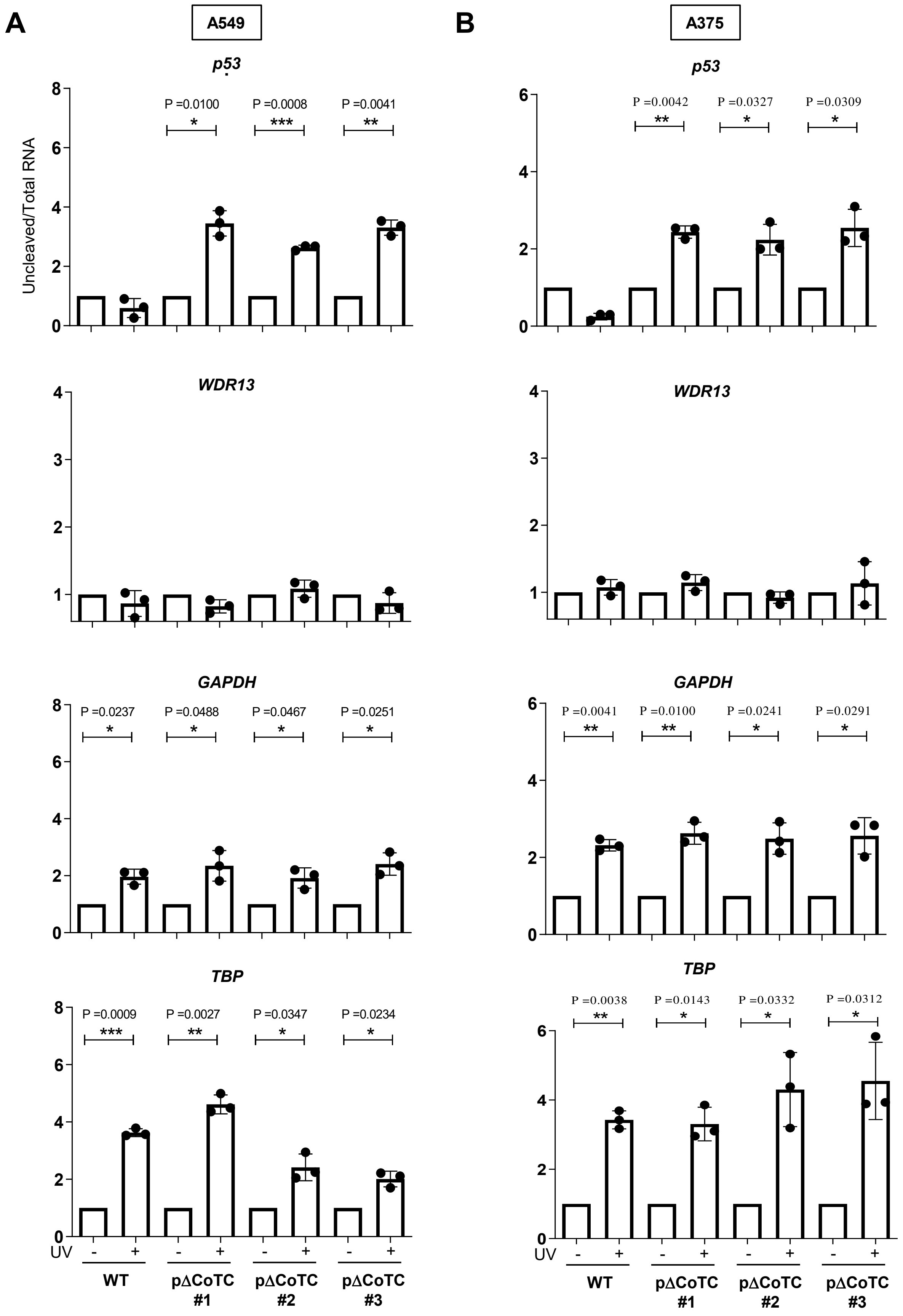

A
A549
B
A375
p53
Uncleaved/Total RNA
P =0.0100
P =0.0008
P =0.0041
*
***
**
P =0.0042
P =0.0327
P =0.0309
WDR13
GAPDH
P =0.0237
P =0.0488
P =0.0467
P =0.0251
P =0.0041
P =0.0100
P =0.0241
P =0.0291
TBP
P =0.0009
P =0.0027
P =0.0347
P =0.0234
P =0.0038
P =0.0143
P =0.0332
P =0.0312
UV
WT
pΔCoTC #1
pΔCoTC #2
pΔCoTC #3

Appendix Figure S5. ***Partial deletion of the CoTC element inhibits p53 PAS cleavage in response to UV .*** RT-qPCR assay on nuclear RNA for assessing the uncleaved/total ratio of p53 pre-mRNA in wild type (WT) and partial CoTC deleted (pΔCoTC) A549 (A) or A375 (B) cells (n=3) treated with or without UV irradiation (40 J/m2). "n" indicates the number of biological replicates for each experiment. All data are presented as the mean ± s.e.m. P values were calculated using two-sided unpaired t-test.

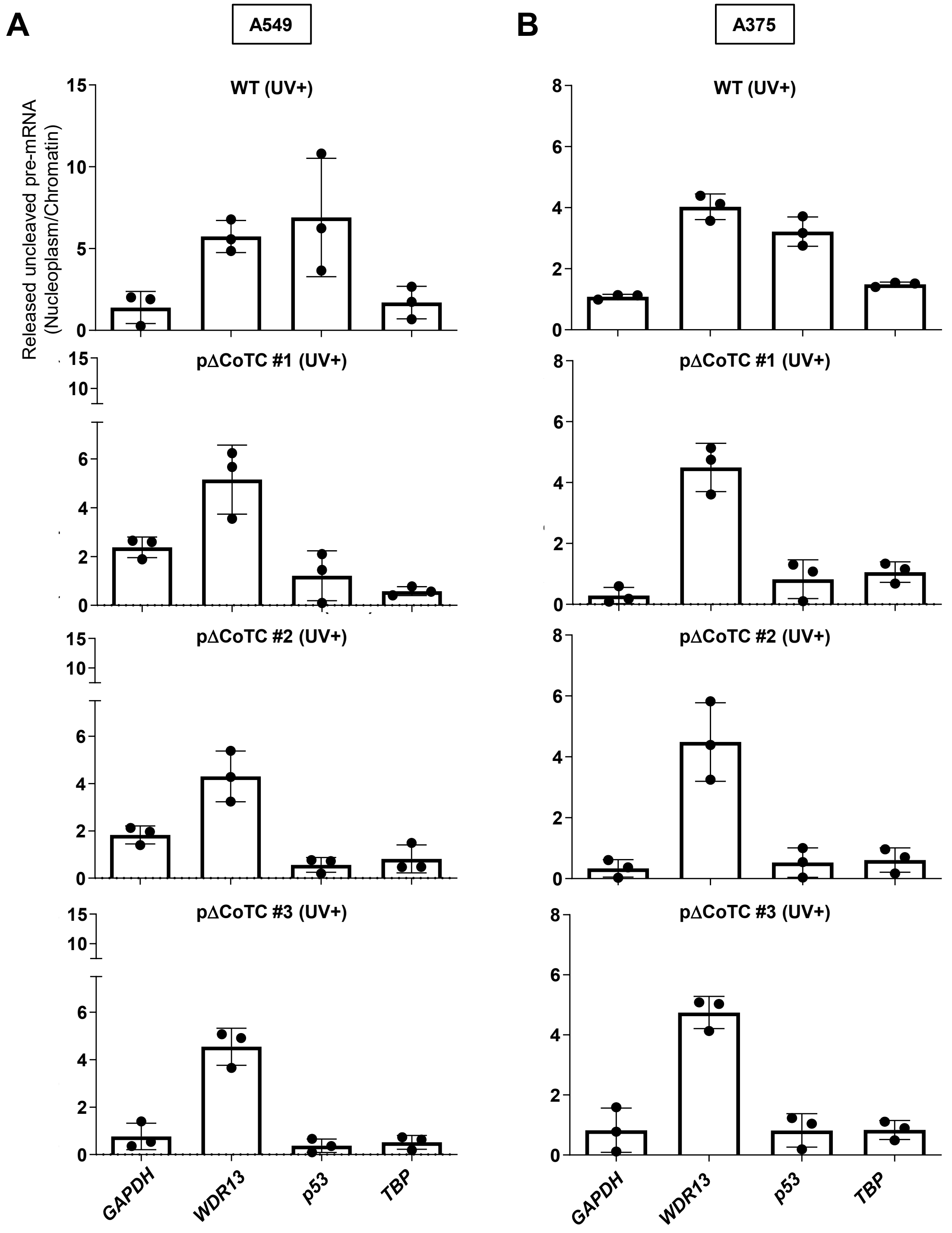

A
A549
B
A375
WT (UV+)
pΔCoTC #1 (UV+)
pΔCoTC #2 (UV+)
pΔCoTC #3 (UV+)
Released uncleaved pre-mRNA (Nucleoplasm/Chromatin)
GAPDH
WDR13
p53
TBP

Appendix Figure S6. ***Partial deletion of the CoTC element inhibits the nucleoplasmic processing of p53 in response to UV.*** RT-qPCR analysis on RNA extracted from nucleoplasm and chromatin fractions. The ratio of uncleaved pre-mRNA (nucleoplasm/chromatin) was calculated to quantify the level of unprocessed *p53, WDR13, GAPDH* and *TBP* pre-mRNAs released in the nucleoplasm compared to the chromatin-bound unprocessed pre-mRNA in wild type (WT) and partial CoTC deleted (pΔCoTC) A549 or A375 cells (n=3) treated with or without UV irradiation (40 J/m2). "n" indicates the number of biological replicates for each experiment. All data are presented as the mean ± s.e.m.

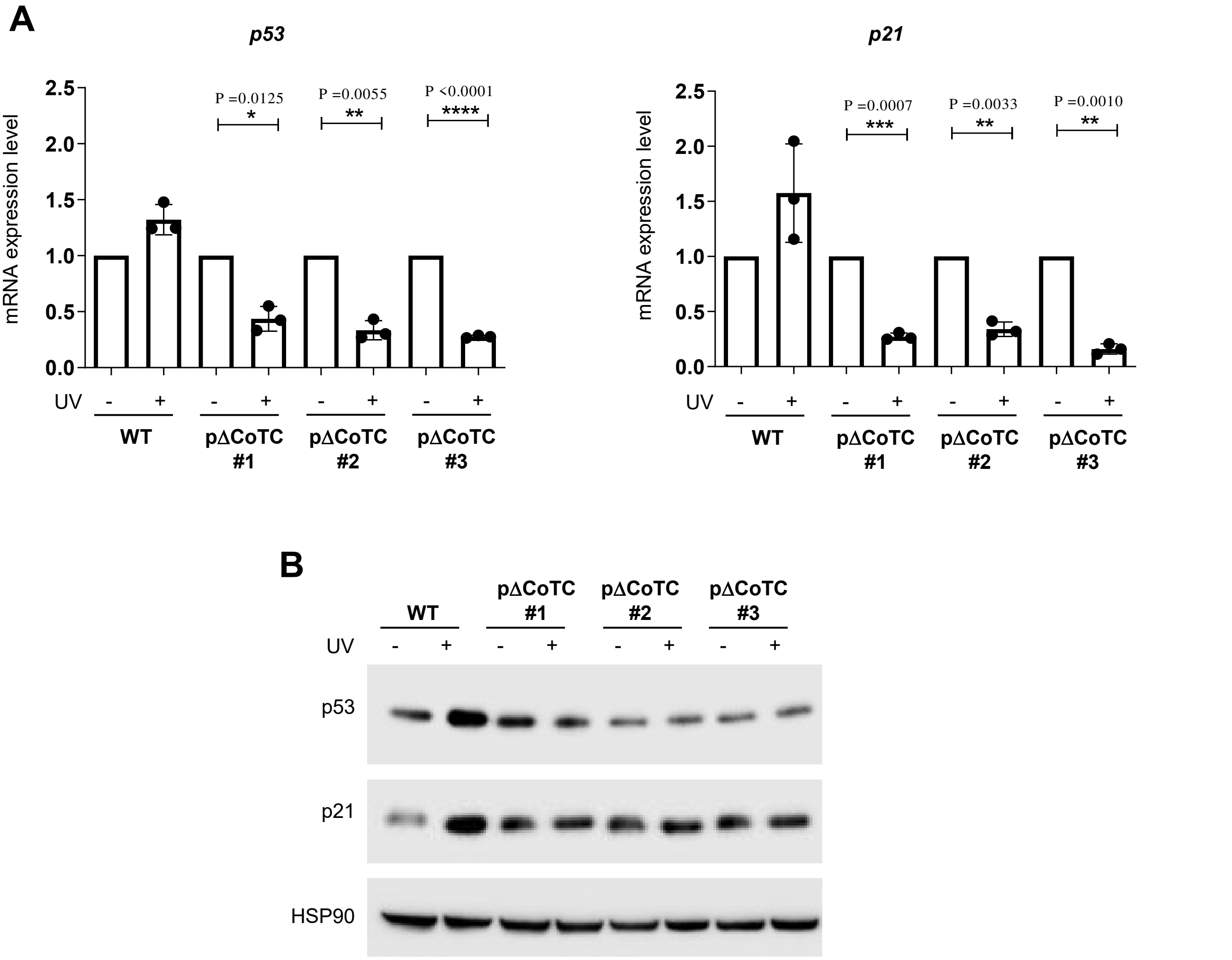

Appendix Figure S7. ***Partial deletion of the CoTC element inhibits the expression of p53 as well as p21 in response to UV***

A. RT-qPCR measuring relative p53 and p21 mRNA levels in wild type (WT) and partial CoTC-deleted (pΔCoTC) A549 cells (n=3) in response to UV treatment (40 J/m2). The expression was normalized to HPRT.

B. Western blot analysis of p53 and p21 expression wild type (WT) and partial CoTC deleted (pΔCoTC) A549 cells (n=3) treated with or without UV irradiation (40 J/m2).

“n” indicates the number of biological replicates for each experiment. All data are presented as the mean ± s.e.m. P values were calculated using two-sided unpaired t-test.

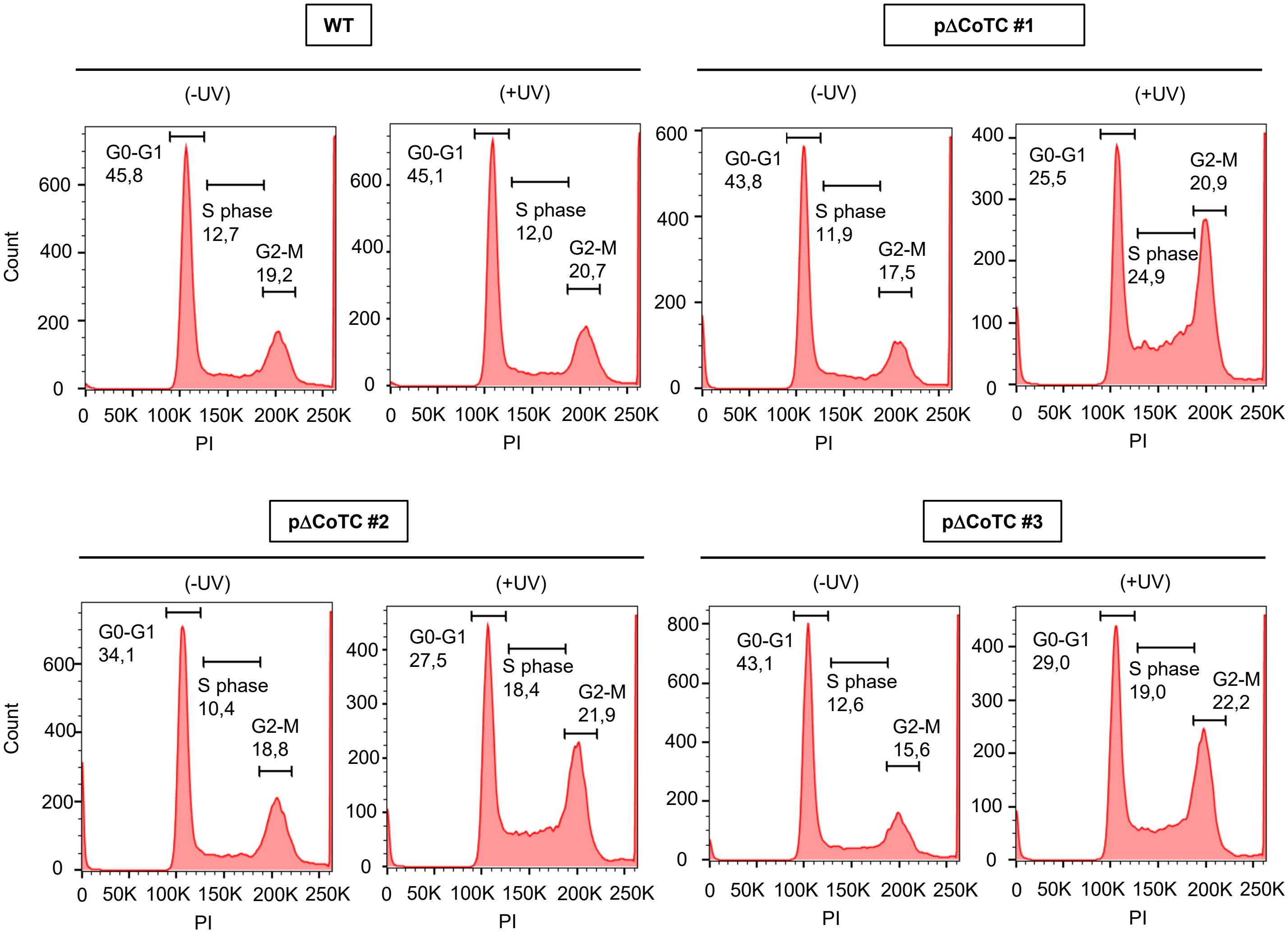

Appendix Figure S8. ***Partial deletion of the CoTC element inhibits cell cycle progression in response to UV.*** Representative flow-cytometry analyses of the cell cycle (DNA content by Propidium Iodide; PI) in wild type (WT) and partial CoTC deleted (pΔCoTC) A549 cells (n=3 biological replicates) treated with or without UV irradiation (40 J/m2).. Indicated: percent of cells in the G0-G1, S and G2/M phases.

A

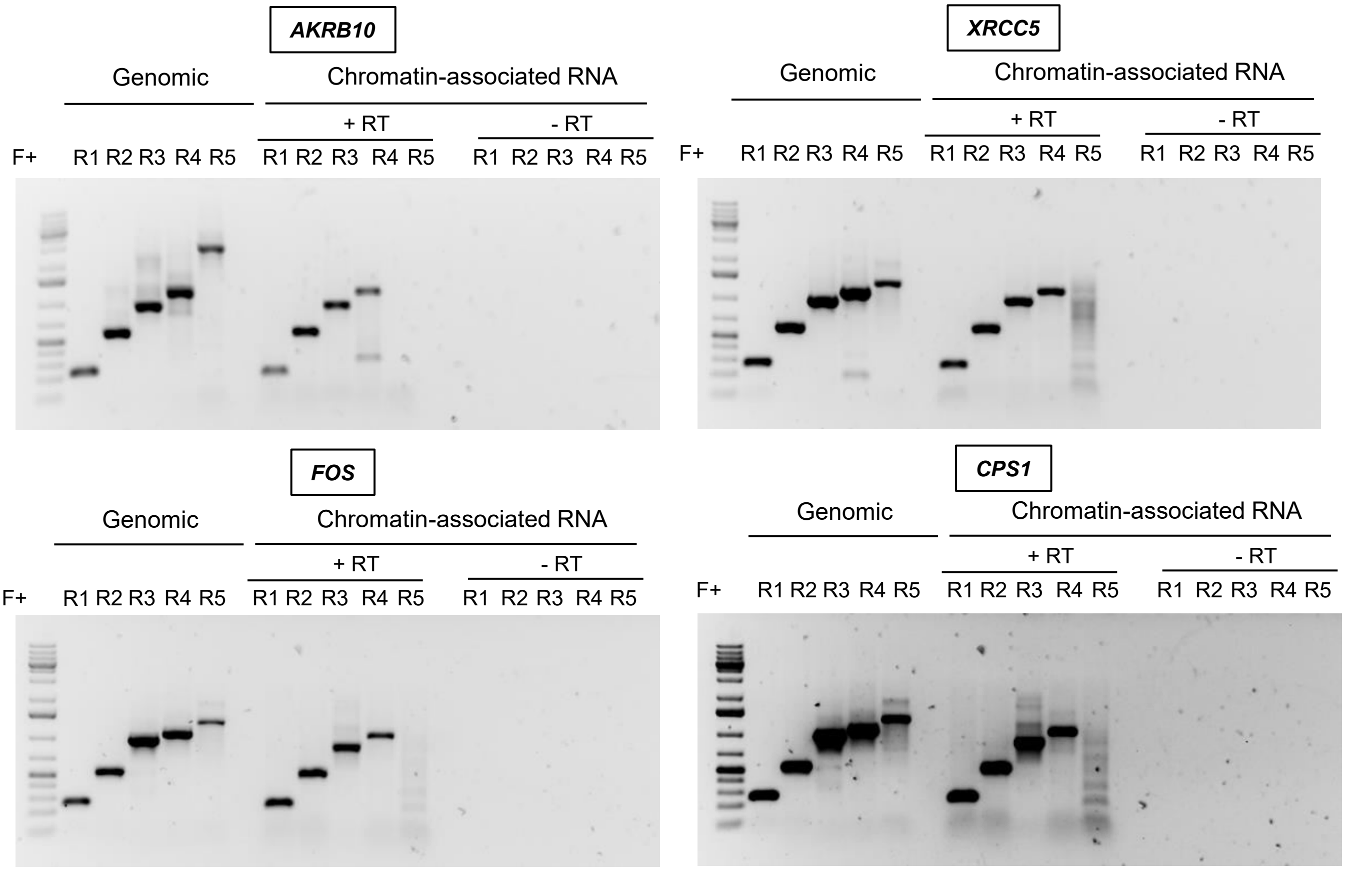
AKRB10
Genomic
Chromatin-associated RNA
+ RT
- RT
F+
R1 R2 R3 R4 R5
R1 R2 R3 R4 R5
R1 R2 R3 R4 R5
XRCC5
Genomic
Chromatin-associated RNA
+ RT
- RT
F+
R1 R2 R3 R4 R5
R1 R2 R3 R4 R5
R1 R2 R3 R4 R5
FOS
Genomic
Chromatin-associated RNA
+ RT
- RT
F+
R1 R2 R3 R4 R5
R1 R2 R3 R4 R5
R1 R2 R3 R4 R5
CPS1
Genomic
Chromatin-associated RNA
+ RT
- RT
F+
R1 R2 R3 R4 R5
R1 R2 R3 R4 R5
R1 R2 R3 R4 R5

B

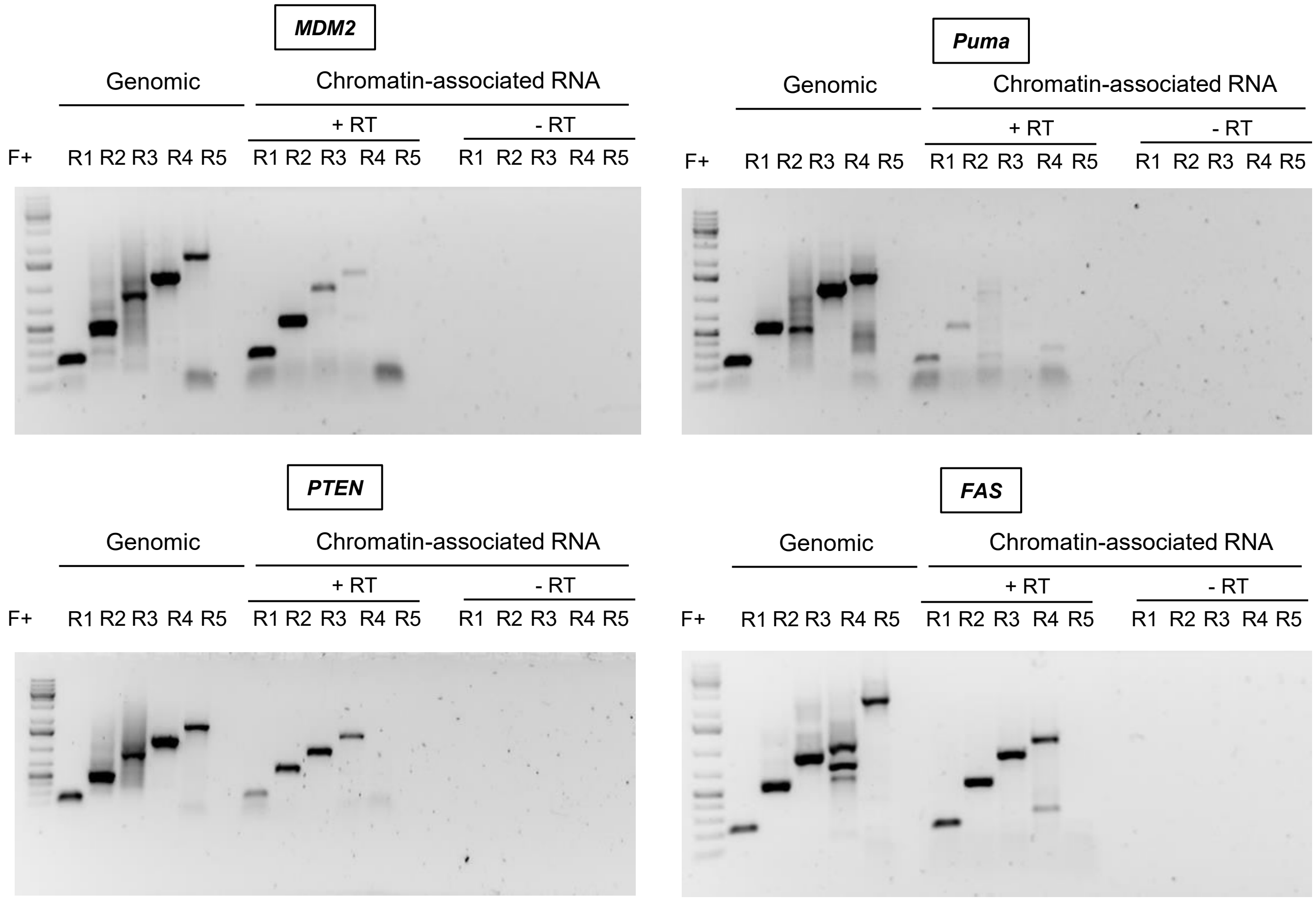
MDM2
Genomic
Chromatin-associated RNA
+ RT
- RT
F+
R1 R2 R3 R4 R5
R1 R2 R3 R4 R5
R1 R2 R3 R4 R5
Puma
Genomic
Chromatin-associated RNA
+ RT
- RT
F+
R1 R2 R3 R4 R5
R1 R2 R3 R4 R5
R1 R2 R3 R4 R5
PTEN
Genomic
Chromatin-associated RNA
+ RT
- RT
F+
R1 R2 R3 R4 R5
R1 R2 R3 R4 R5
R1 R2 R3 R4 R5
FAS
Genomic
Chromatin-associated RNA
+ RT
- RT
F+
R1 R2 R3 R4 R5
R1 R2 R3 R4 R5
R1 R2 R3 R4 R5

C

Basepairs downstream of polyA	100-200	400-600	1000-1200	1500-2000	2500-3000
MDM2	+	+	+	+	-
Puma	+	+	-	-	-
PTEN	+	+	+	+	-
FAS	+	+	+	+	-
AKRB10	+	+	+	+	-
XRCC5	+	+	+	+	-
FOS	+	+	+	+	-
CPS1	+	+	+	+	-

COTC cleavage sites between 1 – 2.5 kb
downstream to the PAS.

D

Chromatin-associated RNA
(RT with oligo dT)

AKRB10 C R1 R2 R3 R4 R5 ***XRCC5*** C R1 R2 R3 R4 R5

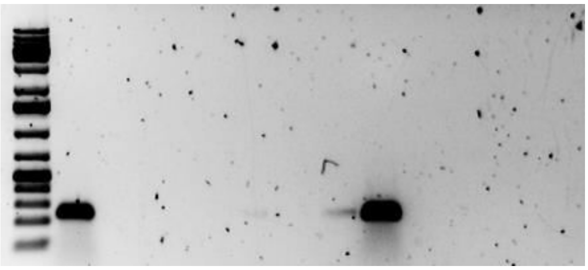

FOS C R1 R2 R3 R4 R5 ***CPS1*** C R1 R2 R3 R4 R5

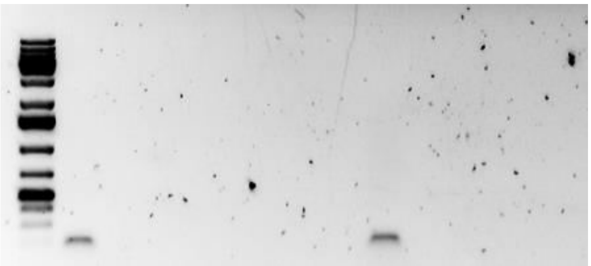

MDM2 C R1 R2 R3 R4 R5 ***Puma*** C R1 R2 R3 R4 R5

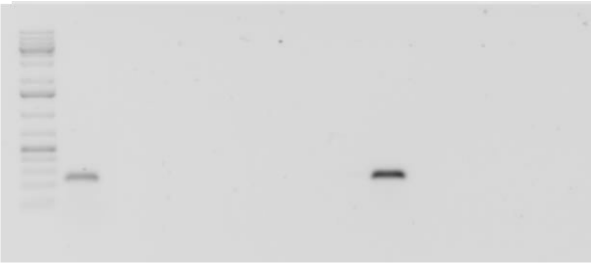

PTEN C R1 R2 R3 R4 R5

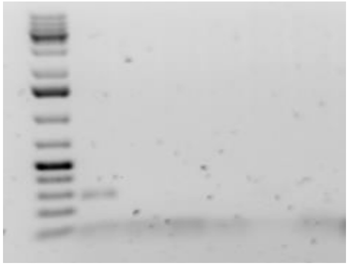

FAS C R1 R2 R3 R4 R5

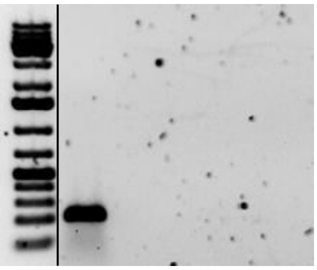

Appendix Figure S9. ***CoTC elements were identified in the 3' flanking regions of candidate genes***

A. PCR analysis of RNA seq candidate gene 3' flanking regions to map the location of CoTC elements. (n=3)

B. PCR analysis of 3' flanking regions in candidate genes from the p53 signaling pathway to map the location of CoTC elements. (n=3)

C. Table to summarize the presence or absence of bands from PCR amplification using primer pairs F/(R1-R5) in 3' flanking regions of candidate genes.

D. PCR analysis of candidate gene 3' flanking region using the same primers employed in the data panel above. Lane 1 is a control PCR amplification of cDNA derived from the reverse transcription of a control mRNA using oligo (dT). Lanes 2-6 are PCR amplification of reverse transcribed candidate gene chromatin-associated pre-mRNA using oligo oligo (dT). Likewise for all genes. (n=3)

"n" indicates the number of biological replicates for each experiment.

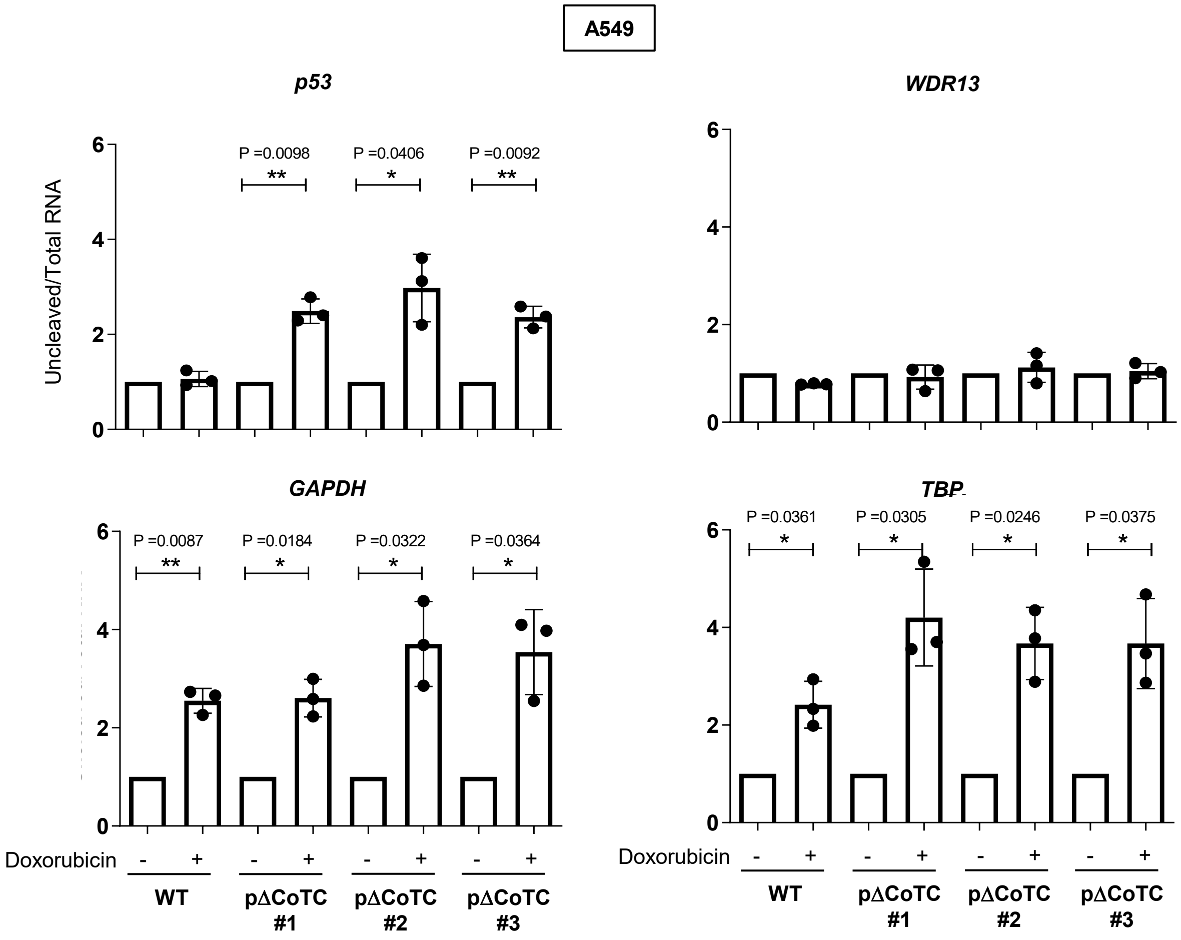

Appendix Figure S10. ***Partial deletion of the CoTC element inhibits p53 PAS cleavage in response to doxorubicin.*** RT-qPCR assay on nuclear RNA for assessing the uncleaved/total ratio of *p53* pre-mRNA in wild type (WT) and partial CoTC deleted (pΔCoTC) A549 cells (n=3) treated with or without doxorubicin (3.5 μM). "n" indicates the number of biological replicates for each experiment. All data are presented as the mean ± s.e.m. P values were calculated using two-sided unpaired t-test.

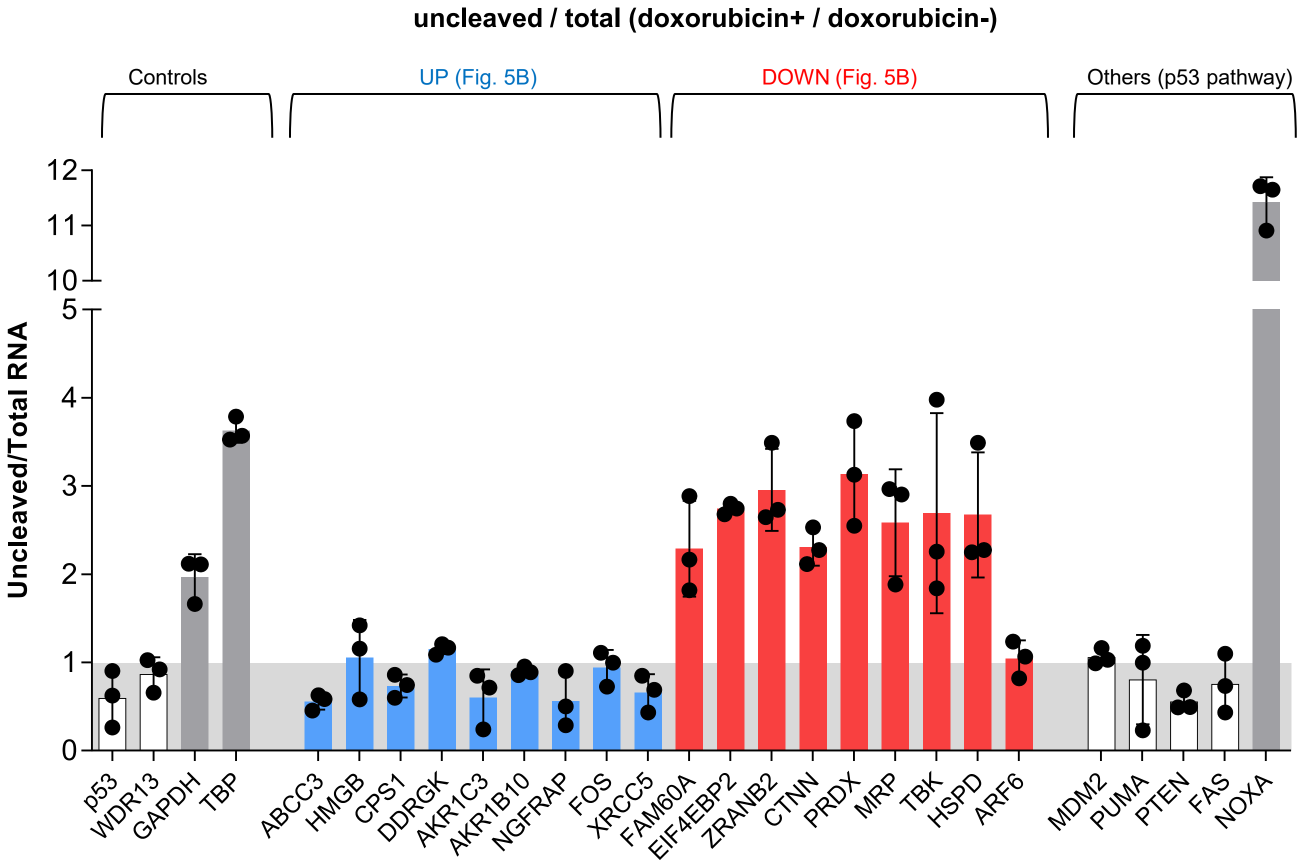

Appendix Figure S11. ***Validation of PAS cleavage inhibition of candidate pre-mRNAs in response to doxorubicin.*** RT-qPCR (uncleaved/total RNA) on nuclear RNA extracted from doxorubicin-treated or untreated A549 cells (n=3), to assess the regulation of 3'end processing of 20 pre-mRNAs randomly selected from the previous RNA-sequencing data. "n" indicates the number of biological replicates for each experiment. All data are presented as the mean ± s.e.m.

Appendix Table S1. ***siRNA sequences for all genes tested***

Gene	siRNA sequence
CPSF160	GCUUUAAGAAGGUCCCUCA
CPSF100	CUCAACUUCUUGAUCAGAU
CPSF73	CCAUAUACUGGUCCCUUUA
CPSF30	GUGCCUAUAUCUGUGAUUU
CstF77	GAAGACUUAUGAACGCCUU
CstF64	GGCUUUAGUCCCGGGCAGA
CstF50	GUCGUAAGUCCGUGCACCA
CFIm68	CUGCAAUUUCUUUAAUUAA
CFIm25	CCUCUUACCAAUUAUACUU
CFIm59	CUCAUCUGCUCGUGUGGAU
CLP1	GCUUAUGUCUCCAAGGACA
Fip1	CGAAUGGGACUUGAAGUUA
PCF11_1	GUACCUUAUGGAUUCUAUU
PCF11_2 (pool)	GAUACAAAUCAGCGACUUA
	GUGUGCAAAUUUAACGAAA
	AAGUUAAGGAAGAACGAAU
	GGAUAAGACCGAUGGCAAA
hnRNP H1	GGUAUUCGUUUCAUCUACA
hnRNP F	GGUGUCCAUUUCAUCUACA
DHX36	GGUGUUCGGAAAAUAGUAA

Appendix Table S2. ***20-mer protospacer sequences for two sgRNA and their reverse complement for the deletion of p53 CoTC***

**sgRNA**	**Sequence**	**Reverse complement**	**PAM**
**sgRNA_A**	CTGTCCTTGCCTCTGTAGAC	GTCTACAGAGGCAAGGACAG	AGG
**sgRNA_B**	CGTGCTGATTAATTTGATTG	CAATCAAATTAATCAGCACG	TGG

Appendix Table S3. ***Modified sgRNA sequences to facilitate cloning*****.** Protospacer sequences and their reverse complements with "CACC" and "AAAC" added for cloning into the pX458/pX459 vector using BbsI restriction enzyme

sgRNA	Sequence	Reverse complement	PAM
**sgRNA_A**	CACCGCTGTCCTTGCCTCTGTAGAC	AAACGTCTACAGAGGCAAGGACAGC	AGG
**sgRNA_B**	CACCGCGTGCTGATTAATTTGATTG	AAACCAATCAAATTAATCAGCACGC	TGG

Appendix Table S4. ***Primer sequences used for all genes tested***

Gene	Primer name	Sequence
Primer sequences		
TP53	Forward	AGGCGATCCACCTGTCTCA
	Reverse R1	TAGCCTGCACTGGCGTTC
	Reverse R2	TGGAGGCTCAGCCTTGCTAA
	Reverse R3	AGTACTGAGCTCCTCAACC
	Reverse R4	GAGTGTTTGGCATTCCCTAGTA
	Reverse R5	GAAGCAGCACAGCACAGCAGAAATAAA
AKRB10	Forward	TCTGCCAACACTGAGGATGT
	Reverse R1	TTGAGCAAGTTCCTCCTCCC
	Reverse R2	GGACAAACAGAAATGTTCCAGAT
	Reverse R3	ACAGGGAGAGAGGGAGAGAG
	Reverse R4	TATCACTGGGCTCTGGGTTG
	Reverse R5	GGTAAGTCTAGCCCTCTGGA
XRCC5	Forward	AGCACCCTCATAAGTCGTCA
	Reverse R1	TGAGCACCTGTATGTCAAGTT
	Reverse R2	GCACAAATAATCCTGCTGCA
	Reverse R3	ACTGGCAAAGGATTAAAACCCA
	Reverse R4	TGTACTCCAGCCTCGGTG
	Reverse R5	CTCTGCCTCCCAAAGTGCT
FOS	Forward	TGTTTGCTTATTGTTCCAAGACA
	Reverse R1	CGTCCCCAGAAGCAGTAGAA
	Reverse R2	GCAGGAAGATTCTAATGCCGA
	Reverse R3	ACGATCAGCCATTATTTGTGC
	Reverse R4	TGAACAGCAAACAGGGATCC
	Reverse R5	TTGAGGTCAGGAGTTCGAGG
CPS1	Forward	AGGGCAGCCTTTGTTACTTT
	Reverse R1	AGCAAGGGAGGGACAAGAAA
	Reverse R2	TGGTAATCAATTGACTGTGAGGT
	Reverse R3	ATGGTGATGGTGGTTGTGGT
	Reverse R4	CAGCCTGCTCACTTTTAGTCA
	Reverse R5	CATTGTTCAAGAGGCTGTGGA
MDM2	Forward	AGGTAGATATCTGAAAGCACCA
	Reverse R1	TGTTTCAGTACCACTCCTCTCT
	Reverse R2	GGAGGTTGAGGCTGTAGTGA
	Reverse R3	CTCACGCCTGTAATCCCAGT
	Reverse R4	TGGGGAGGTGTGAACCAAAA
	Reverse R5	CTTCAAGGTGGAGTAGGGGT
Puma	Forward	CGCTGCTGTAGATACCGGAA
	Reverse R1	GCCTTTCTTCTGATGGAGCC
	Reverse R2	GGCTTGATCATCGCTCACTG
	Reverse R3	CGTCTCGATCTCCTGACCTC
	Reverse R4	GCTCGCTGTAACCTTTATCTCC
	Reverse R5	CGTACAGTGGTGCAATCTCG
PTEN	Forward	AATGCCTCATCCCAATCAGAT
	Reverse R1	TTCTGAACTAGCAACAGCACT
	Reverse R2	TGTTGTTGTGATGGGGAAGT
	Reverse R3	AGCCACTGAATTCGAAAGGA
	Reverse R4	ACGCGGTAATTTTCAGAGCT
	Reverse R5	GCCTCACTTCATTCCACACA
FAS	Forward	TTTGCCCCTTGTGTTTGGAA
	Reverse R1	TGTGCTGTTTGGAAGAGGTC
	Reverse R2	GGAACCCTAAGCAAAGCACA
	Reverse R3	CCACCACAAAGAGAACCAGG
	Reverse R4	AGCAGACATAATCAACAGCAACA
	Reverse R5	TCCTAAAATGCAACATACGGAGA
PCF11	Forward total	AGCCGAAAAGTCACTCATAGAC
	Reverse total	GCCTCTTGAGTTTTGAGCAC
TP53	Forward total	AGGCGATCCACCTGTCTCA
	Reverse total	CAGATGTGCTTGCAGAATGT
	Forward uncleaved	AGGCGATCCACCTGTCTCA
	Reverse uncleaved	TAGCCTGCACTGGCGTTC
TBP	Forward total	GGAAGGGGCATTATTTGTG
	Reverse total	GCCCAGATAGCAGCACGGTA
	Forward uncleaved	GCAGGACAGAATATATGTGTTAATG
	Reverse uncleaved	CAGTATGATCACATGACTCTTACAAGG
	Forward total	CATGGTCATCGTCTGGAGGC
	Reverse total	TAAGAGGGGTGGGATGGAGG

WDR13	Forward uncleaved	CATTCATGCATCGACGGATTC
	Reverse uncleaved	TAGAACAGTTCCTGGCACAC
GAPDH	Forward total	CCAAGGAGTAAGACCCCTGG
	Reverse total	GTACATGACAAGGTGCGGC
	Forward uncleaved	TACCCTGTGCTCAACCAGTTA
	Reverse uncleaved	CAGCTTCCTGTAGCACTCAA
ABCC3	Forward total	AACAGAAGACAGCTGCTGGG
	Reverse total	AATGGATTCAGGCAGCACCC
	Forward uncleaved	CAGTAGTCTTTTTGCACTTGTTCAC
	Reverse uncleaved	GTAGAAAGTCTTCCTCTTGGCCT
HMGB1	Forward total	TCGTCCCATCACAGTGTTGTT
	Reverse total	CTCGGGTACACAGGACACAC
	Forward uncleaved	GCGCCCATGTAACACAAACT
	Reverse uncleaved	TCCTACAATGTCTGAGCAATGG
CPS1	Forward total	TTCCCTTAAGACGATGGATTCTG
	Reverse total	TGTAGAAGGAATGGTGTCCTTG
	Forward uncleaved	AGGGCAGCCTTTGTTACTT
	Reverse uncleaved	AAACCAGATTCAACTGCATTACC
DDRGK1	Forward total	TGGTGTGGCTTGGTGTG
	Reverse total	AACAGGACTTCACCAGCTTC
	Forward uncleaved	AAATAGCCTGTTGCACATTTACTC
	Reverse uncleaved	TTAACAGAGATGTGGCCCAAG
AKR1C3	Forward total	CTGAGTCCATAGGCCAGAAAG
	Reverse total	ACACTACAGAACAGAGTAGGTAAAG
	Forward uncleaved	CCTACTCTGTTCTGTAGTGTGTG
	Reverse uncleaved	CCCTGTTGAGCCAGAAGAAA
AKR1B10	Forward total	GACGAGAATCGAGGTGCTGT
	Reverse total	TCAAGCCATGCTTTTCTGTGAT
	Forward uncleaved	GCGATCGATGGTCATCCTCTT
	Reverse uncleaved	GAAGGCAAGCTGTGAGAGCA
NGFRAP	Forward total	CCATGTGTCAAGTGGGTCTT
	Reverse total	CCATGCAAATGGGTGAAACTAC
	Forward uncleaved	CACTAGAGTGTTAATTGGTGAACAT
	Reverse uncleaved	ATCTCCTGACCTCGTGATCT
FOS	Forward total	TTGTTGAGGTGGTCTGAATGT
	Reverse total	CTTGGAACAATAAGCAAACAATGC
	Forward uncleaved	AGTTGAATGCGACCAACCT
	Reverse uncleaved	GTCCTCTTTGATAAGGGATCAGAC
XRCC5	Forward total	TTGTGGATGGTGTCTCCTTTAC
	Reverse total	CACCAAAGAGGAACTGGAACT
	Forward uncleaved	GCTGAGAATTGAACACCCTTATC
	Reverse uncleaved	GATGTCCTAGAAGCCCAAAGTA
FAM60A	Forward total	GCTGCAGTATTGGTGGTAGAA
	Reverse total	CAGTACATCCTACAGGCAAAGAG
	Forward uncleaved	GTACTGTATGTAGTCATGCACTTTG
	Reverse uncleaved	CCTGTCAAACAAAGCCACAA
EIF4EBP2	Forward total	TGTCTCCCATGATGTGTTGTT
	Reverse total	CACACAGGACTGCCTCAAG
	Forward uncleaved	TTCTGGTGAAATCCTGCTAAGG
	Reverse uncleaved	AGTGTGGAGAACTGACAGATAAAG
ZRANB2	Forward total	GCTGTACTAAGCAAATGCAAGG
	Reverse total	TGCTTGACTCACAGGCTTTAT
	Forward uncleaved	ATTCCAAAGCCATTATCACTGC
	Reverse uncleaved	TCAGGAAGCACACTACGATATG
CTNNB1	Forward total	GTATGGGTAGGGTAAATCAGTAAGAG
	Reverse total	TCTCTTGAAGCATCGTATCACAG
	Forward uncleaved	CTGTGATACGATGCTTCAA
	Reverse uncleaved	ACCACCCTCACAAACCATTTA
PRDX6	Forward total	TTCCGATGATGTGTACATGAAAGA
	Reverse total	AAATAGCAACCCACTGCAAGA
	Forward uncleaved	GGGTCAGAGAATTCTGTTGTCATA
	Reverse uncleaved	CACGTTCTTCAGCTGTTCCT
MRPL32	Forward total	GGAAGATTCTTTATGTTGTTGTGCT
	Reverse total	AATCCATTGAGCCTTTGGATAAAC
	Forward uncleaved	CCAAAGGCTCAATGGATTATGT
	Reverse uncleaved	AAAGGCACTGGCAAACAAA
TBK1	Forward total	CAGAACCGCACCACTGTTA
	Reverse total	GGATACAAGGATAACTGGGATCTG
	Forward uncleaved	AGAGTTCATGTGTTTCTTTGTATCC
	Reverse uncleaved	TTGTCCCTAGATCCAATATTCTGAG
	Forward total	ACCAGTGTACTGCTTTCAACT
	Reverse total	AAGGCTGCTTAACTTCTCATCT

HSPD1	Forward uncleaved	GATGAGAAGTTAAGCAGCCTTTC
	Reverse uncleaved	CTCCCAAGTAGCTGGGATTA
ARF6	Forward total	GAAACACAGCAGTTCTTGGTAAAG
	Reverse total	AGCCATCTACAGCAAGTGATAAG
	Forward uncleaved	ACTATGTTGCAAGTCTGTTTCATC
	Reverse uncleaved	CCACTGTGGGCTAAGTTTACTA
MDM2	Forward total	CGCTTTATGGGTGGATGCTG
	Reverse total	ATTGAAAGCTGGCTACATGGT
	Forward uncleaved	CACCAGCACTTGGAAGGTGT
	Reverse uncleaved	GAGTACAGCAATCATTTCAGATGC
PUMA	Forward total	GAGATTTTGGCTGAAGCCGC
	Reverse total	CAGTATCTTACAGGCTGGGC
	Forward uncleaved	GCTGCTGTAGATACCGGAATGA
	Reverse uncleaved	AGGGAAGGCAAGCAGAAAGA
PTEN	Forward total	AGCAGTGGCTCTGTGTGTAA
	Reverse total	CATCTGATTGGGATGAGGCA
	Forward uncleaved	TCTTGTCATTGTGTGGGTGT
	Reverse uncleaved	AGGCTTTGAAGGACAGCAGG
FAS	Forward total	AGCAGATACCTGGAACCACC
	Reverse total	TTATAATTCCAAACACAAGGGGC
	Forward uncleaved	AGCAGATACCTGGAACCACC
	Reverse uncleaved	GAGTACAGCAATCATTTCAGATGC
NOXA	Forward total	AGGTTGTAGTCACTTTAGATGGAA
	Reverse total	TACCAGATGGTAAAATAGCTGCCT
	Forward uncleaved	AAGTTGATACTGTGGCAGTAAAC
	Reverse uncleaved	GTCTGCTGATGGAAATCAGTTAAA
