# Supplementary material for: Post‐transcriptional polyadenylation site cleavage maintains 3′‐end processing upon DNA damage
Source: EMBO J. 2023 Feb 10;42(7):e112358. doi: 10.15252/embj.2022112358 (PMC10068322; doi:10.15252/embj.2022112358)
Supplement: Supplementary file 2 — Expanded View Figures PDF [file EMBJ-42-e112358-s005.pdf]

# Expanded View Figures

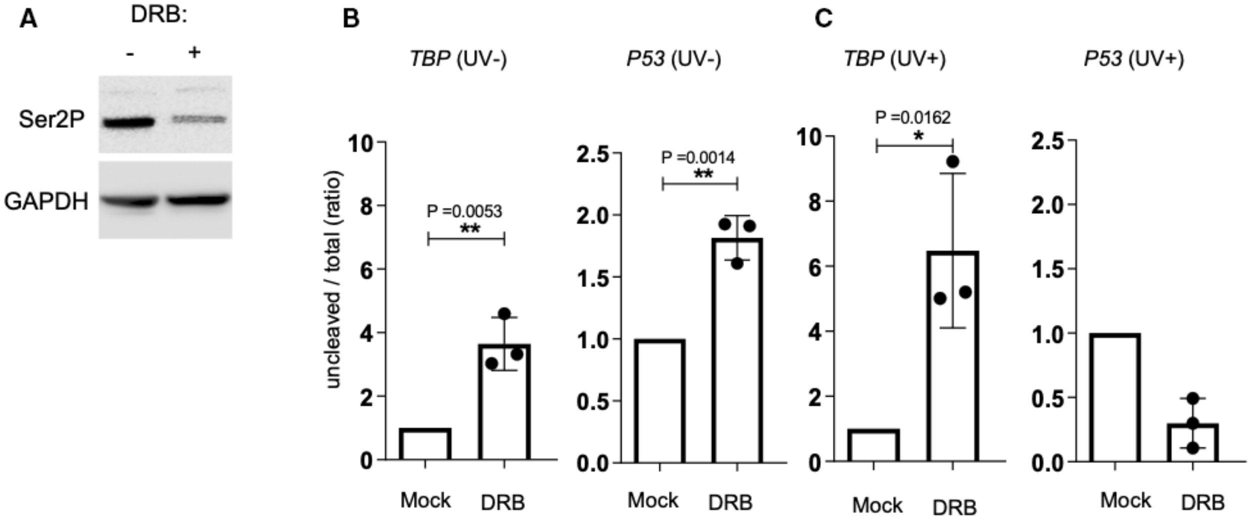


**Figure EV1. The Pol II Ser[2] kinase (CDK9) inhibitor DRB has no impact on *p53* 3′-end processing in UV-treated cells.**

A Western Blot analysis of the CTD phospho-Ser2 expression in A549 cells treated with DRB (50 μM) for 24 h prior to UV irradiation (40 $J/m^2$; *n* = 3).
B RT–qPCR to assess the efficiency of p53 and TBP pre-mRNA 3′-end processing in A549 cells (*n* = 3) treated with DRB (50 μM) for 24 h without UV treatment.
C RT–qPCR to assess the efficiency of p53 and TBP pre-mRNA 3′-end processing in A549 cells (*n* = 3) treated with DRB (50 μM) for 24 h prior to UV irradiation (40 $J/m^2$)

Data information: "*n*" indicates the number of biological replicates for each experiment. All data are presented as the mean ± s.e.m. *P*-values were calculated using a two-sided unpaired *t*-test.
Source data are available online for this figure.

 © 2023 The Authors

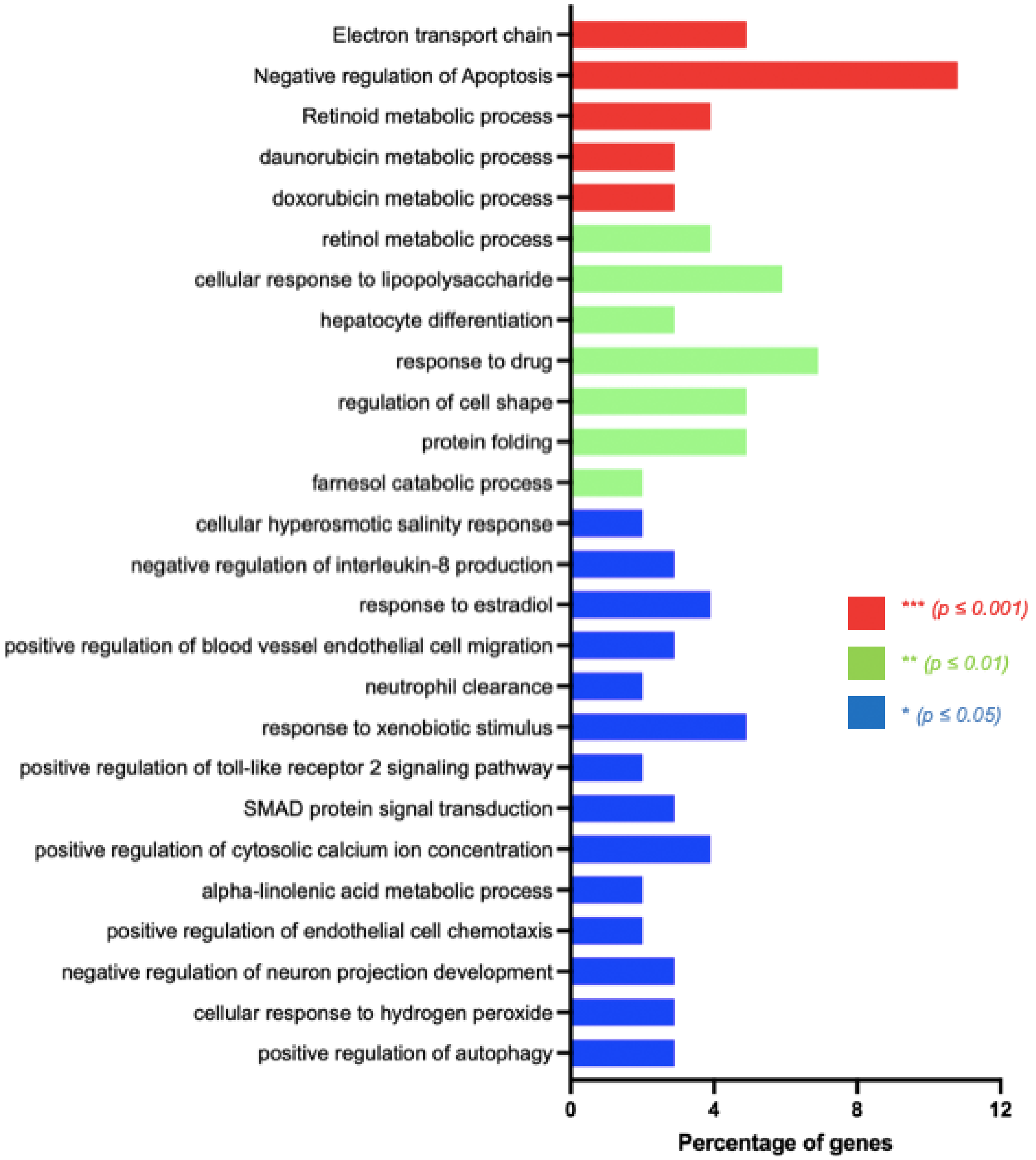


**Figure EV2. Gene ontology (GO) analysis of the 108 pre-mRNAs with a more efficient 3′-end processing in UV-treated compared with untreated cells.**

Data are obtained from RNA-sequencing analyses. The bar chart shows the GO terms for biological processes, ranked by *P*-values, calculated by the functional enrichment analysis tool DAVID.

© 2023 The Authors

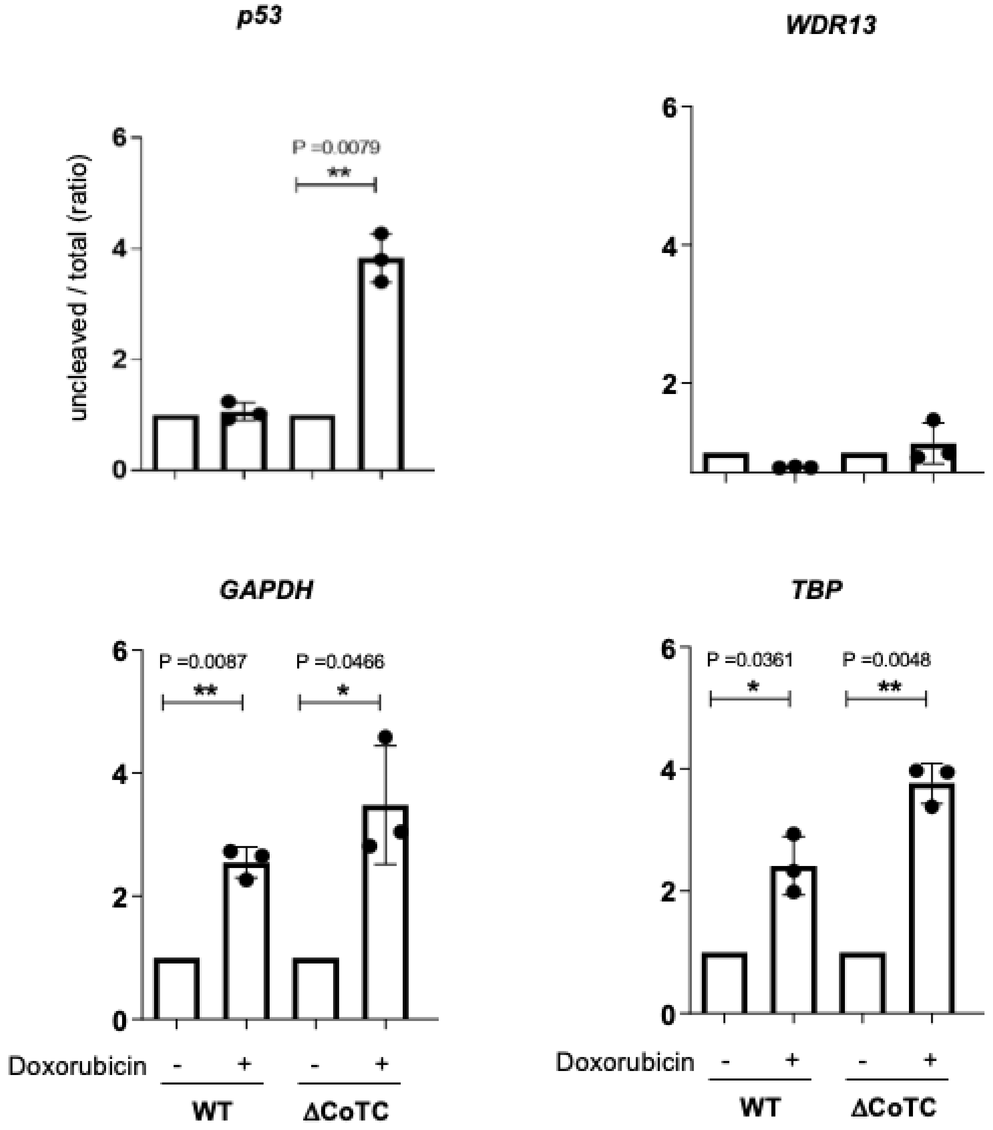


**Figure EV3. The *p53* CoTC region is required for the maintenance of *p53* pre-mRNA 3′-end processing upon doxorubicin treatment.**

RT–qPCR assay on nuclear RNA for assessing the uncleaved/total ratio of *p53* pre-mRNA in wild type (WT) and CoTC-deleted (ΔCoTC) A549 cells treated with or without doxorubicin (3.5 μM). ($n$ = 3); *P*-values were calculated using a two-sided unpaired *t*-test.

Source data are available online for this figure.

 © 2023 The Authors
