# Supplementary figures and images for: Post‐transcriptional polyadenylation site cleavage maintains 3′‐end processing upon DNA damage
Source: EMBO J. 2023 Feb 10;42(7):e112358. doi: 10.15252/embj.2022112358 (PMC10068322; doi:10.15252/embj.2022112358)

## Slide 1
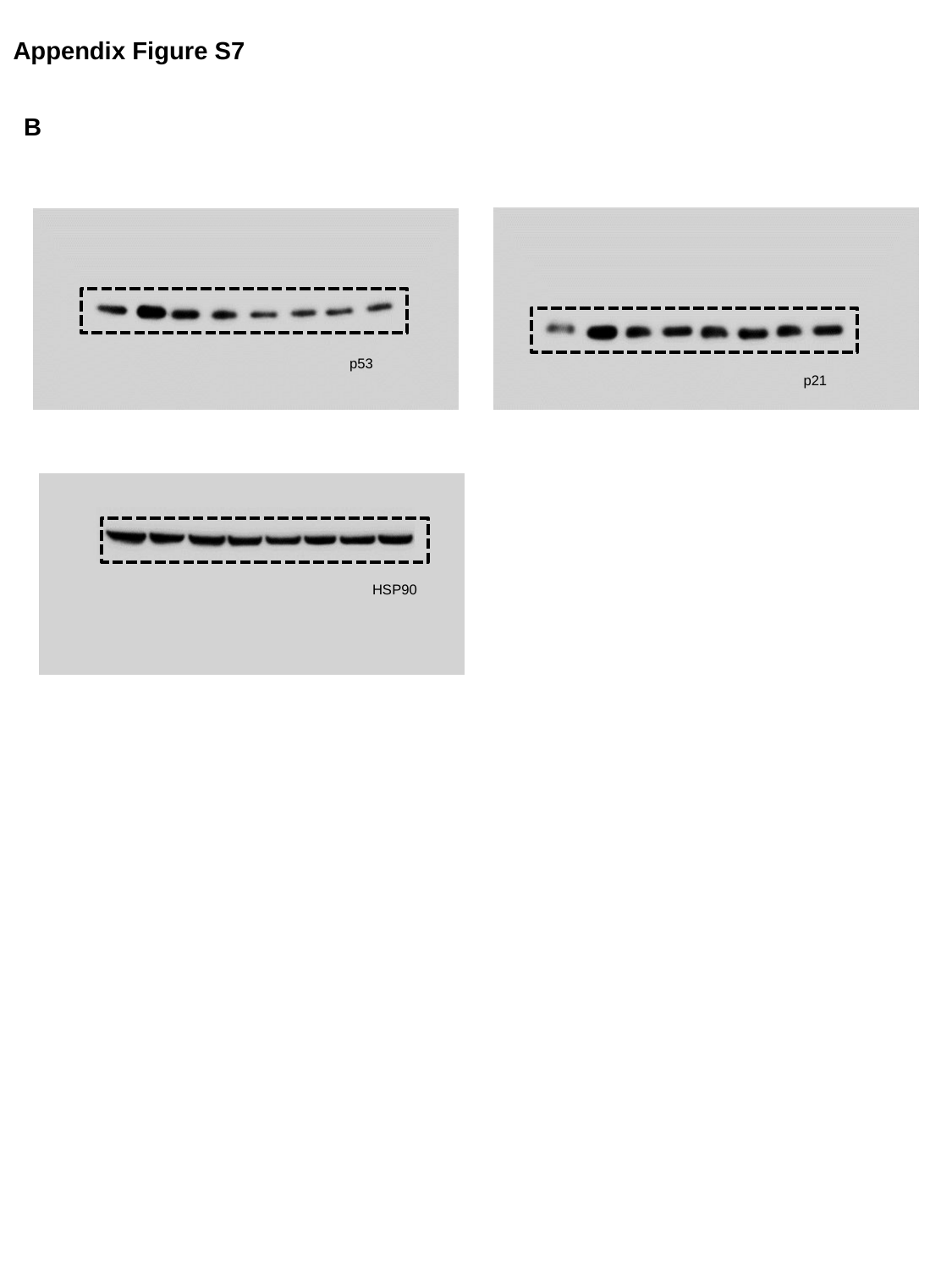

Appendix Figure S7
B
p53
p21
HSP90

Supplement: Supplementary file 4 — Source Data for Expanded View and Appendix [file EMBJ-42-e112358-s007.zip › Source_Data_Appendix/EMBOJ-2022-112358_SourceDataForAppendixFigure S7B.pptx]

## Slide 1
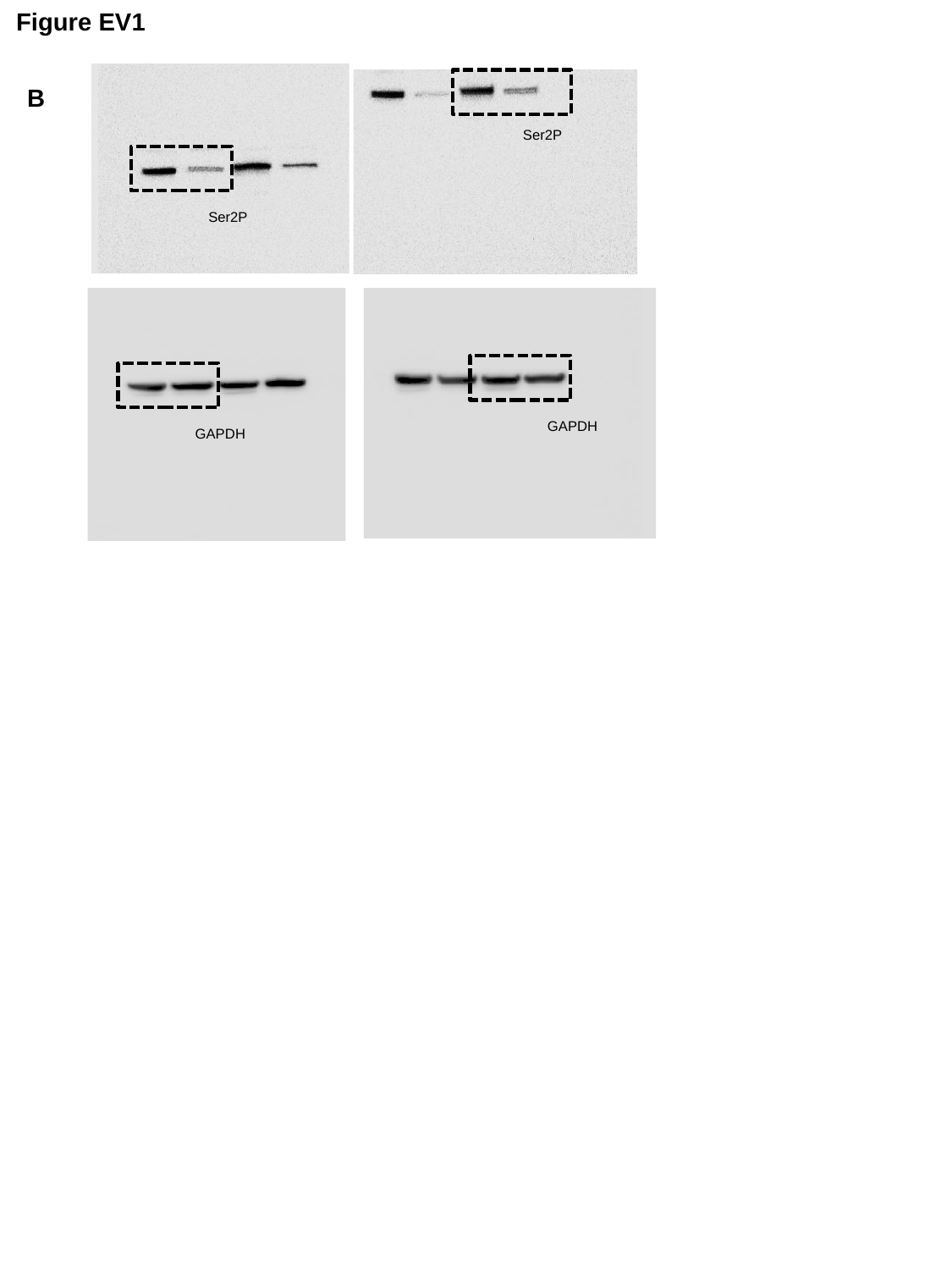

Figure EV1
B
Ser2P
Ser2P
GAPDH
GAPDH

Supplement: Supplementary file 4 — Source Data for Expanded View and Appendix [file EMBJ-42-e112358-s007.zip › Source_Data_EV_Figures/EMBOJ-2022-112358_SourceDataForFigure EV1A.pptx]

## Slide 1
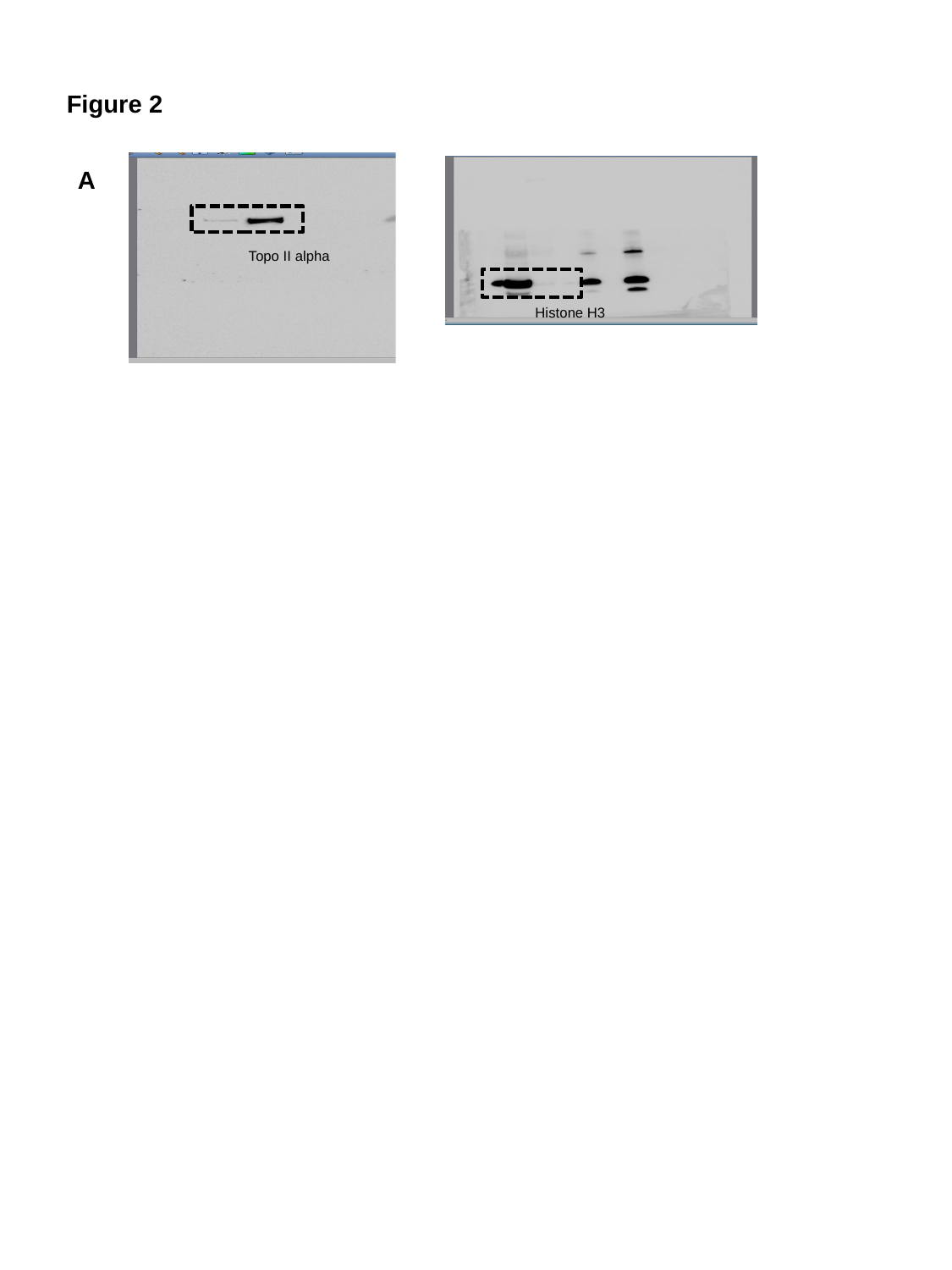

Figure 2
A
Topo II alpha
Histone H3

Supplement: Supplementary file 7 — Source Data for Figure 2 [file EMBJ-42-e112358-s010.zip › EMBOJ-2022-112358_SourceDataForFigure 2A.pptx]

## Slide 1
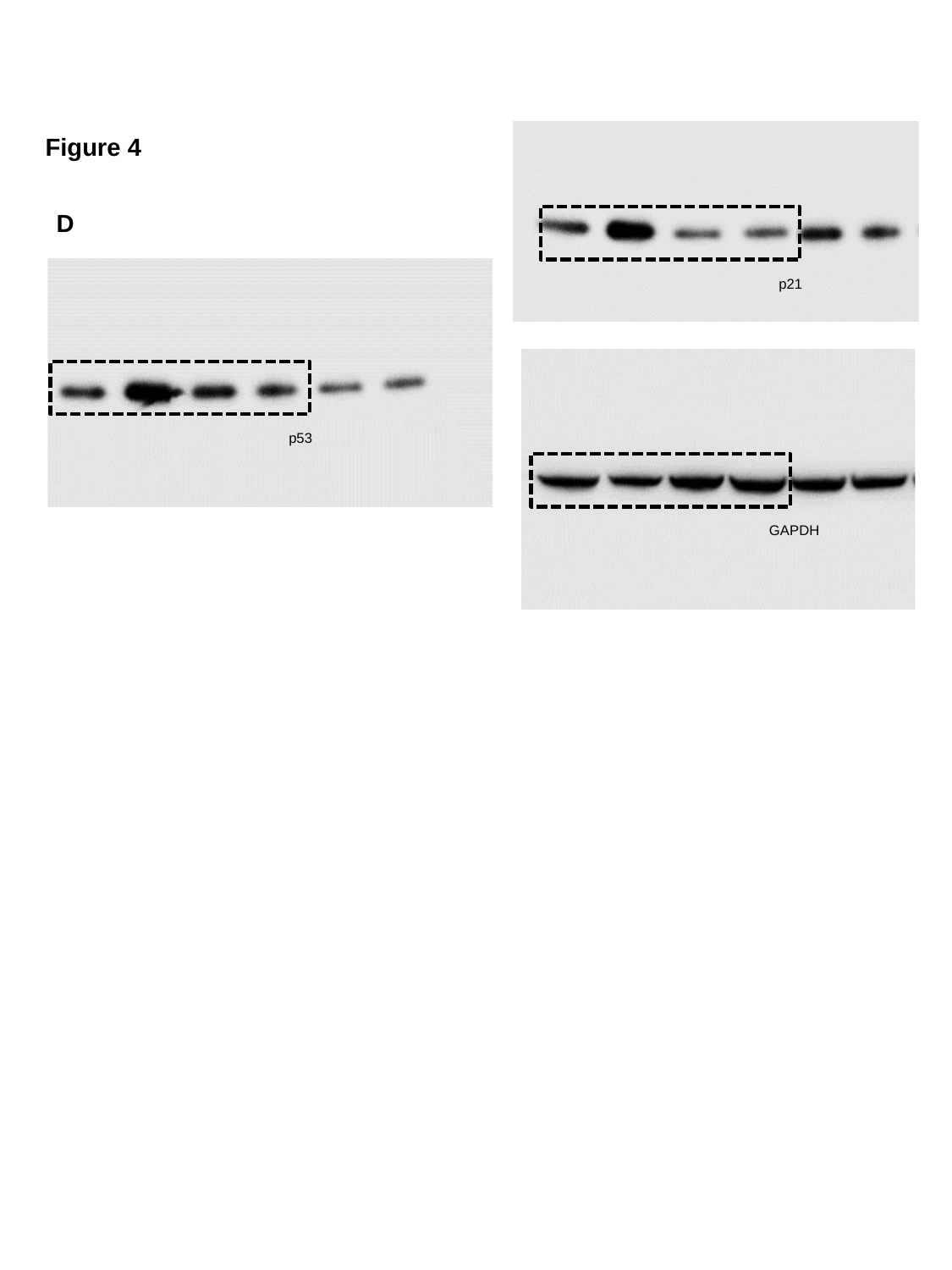

Figure 4
D
p21
p53
GAPDH

Supplement: Supplementary file 8 — Source Data for Figure 4 [file EMBJ-42-e112358-s003.zip › EMBOJ-2022-112358_SourceDataForFigure 4D.pptx]
