# Supplementary material for: Post‐transcriptional polyadenylation site cleavage maintains 3′‐end processing upon DNA damage
Source: EMBO J. 2023 Feb 10;42(7):e112358. doi: 10.15252/embj.2022112358 (PMC10068322; doi:10.15252/embj.2022112358)
Supplement: Supplementary file 6 — Source Data for Figure 1 [file EMBJ-42-e112358-s002.zip › EMBOJ-2022-112358_SourceDataForFigure 1A-B.pptx]

## Slide 1
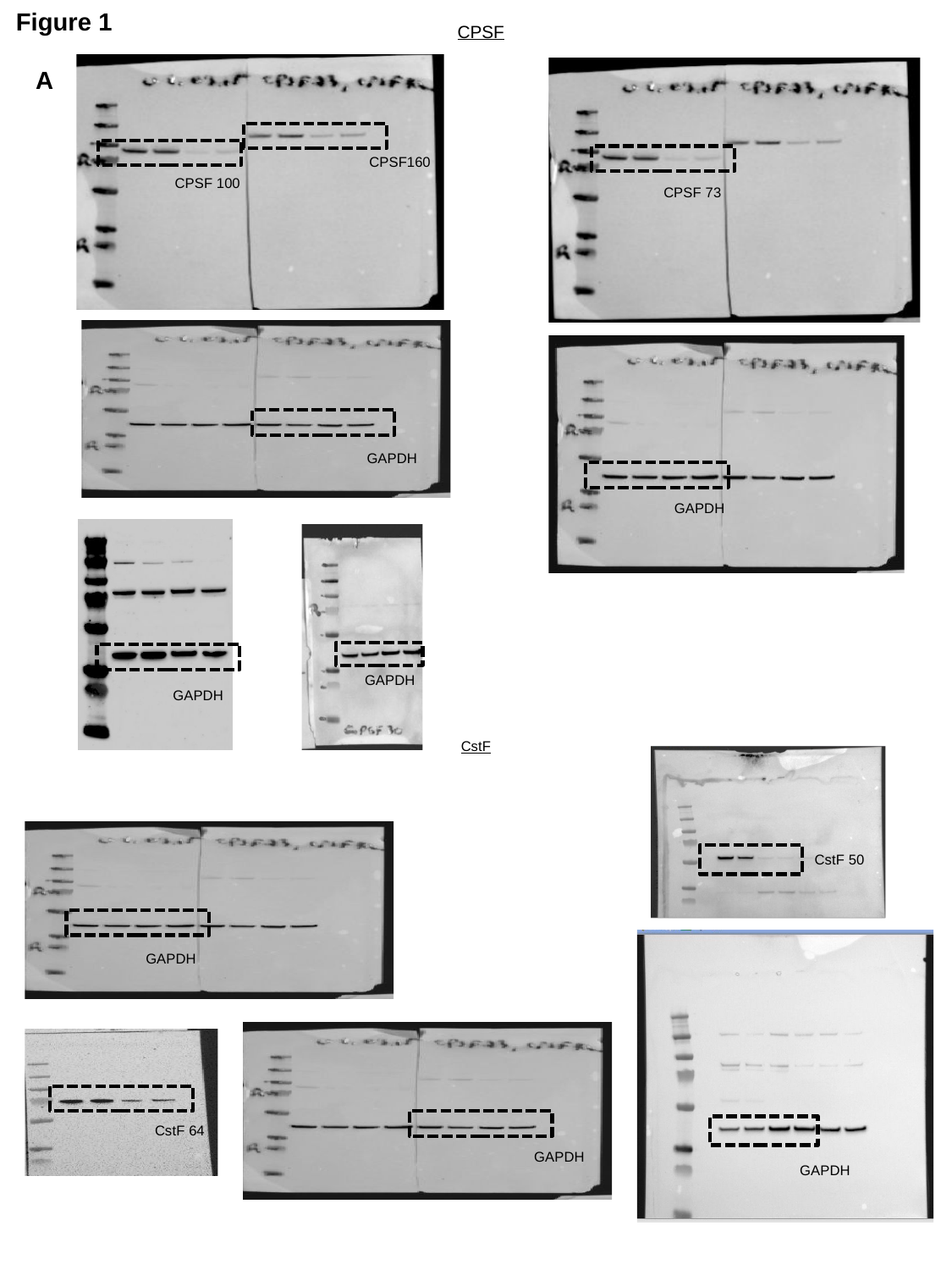

Figure 1
CPSF
A
CPSF160
CPSF 100
CPSF 73
GAPDH
GAPDH
GAPDH
GAPDH
CstF
CstF 50
GAPDH
CstF 64
GAPDH
GAPDH

## Slide 2
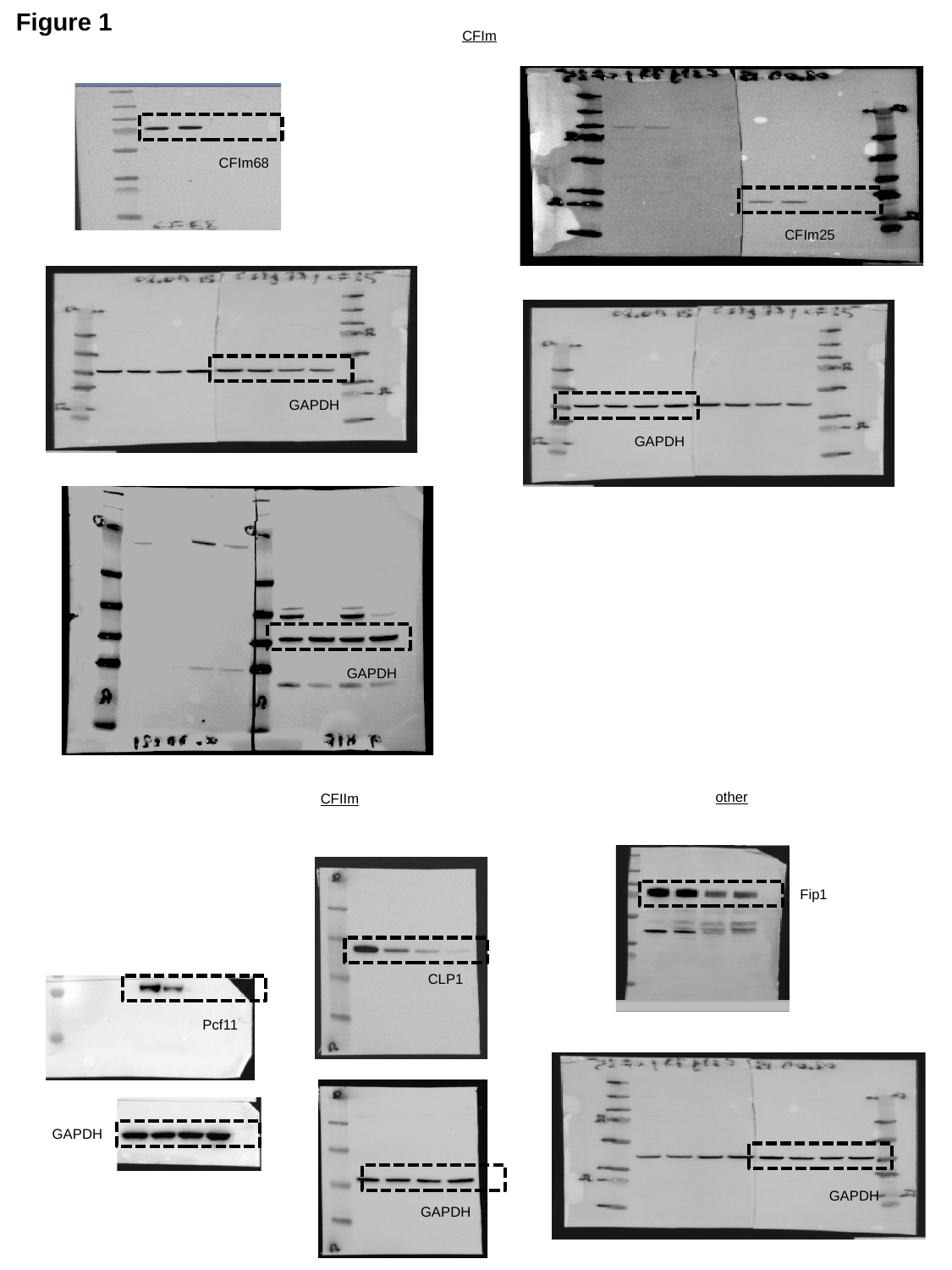

Figure 1
CFIm
CFIm68
CFIm25
GAPDH
GAPDH
GAPDH
other
CFIIm
Fip1
CLP1
Pcf11
GAPDH
GAPDH
GAPDH

## Slide 3
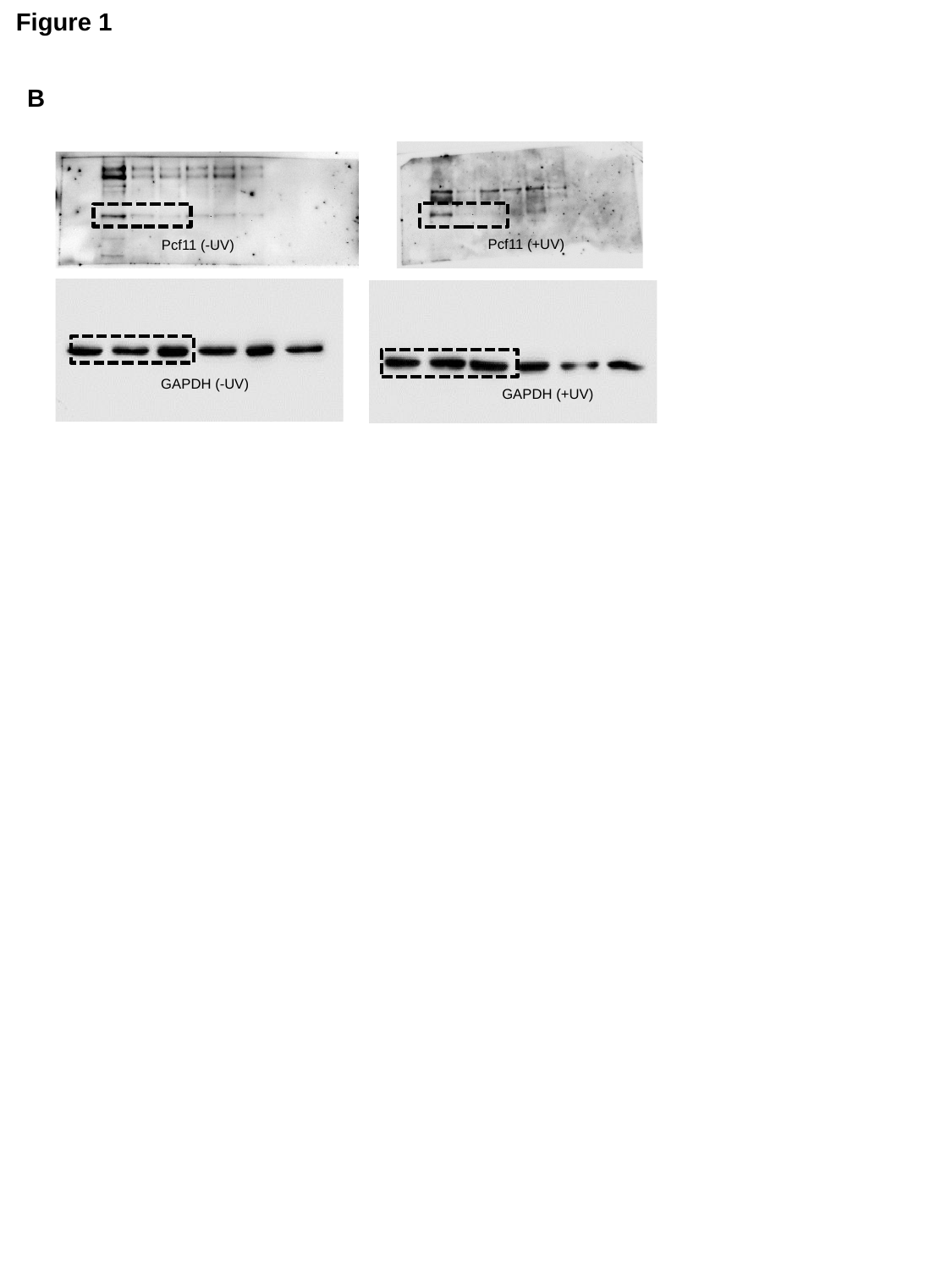

Figure 1
B
Pcf11 (+UV)
Pcf11 (-UV)
GAPDH (-UV)
GAPDH (+UV)
