# Supplementary material for: Post‐transcriptional polyadenylation site cleavage maintains 3′‐end processing upon DNA damage
Source: EMBO J. 2023 Feb 10;42(7):e112358. doi: 10.15252/embj.2022112358 (PMC10068322; doi:10.15252/embj.2022112358)
Supplement: Supplementary file 10 [file EMBJ-42-e112358-s008.pdf]

THE EMBO JOURNAL

Post-transcriptional polyadenylation site cleavage maintains 3'-end processing upon DNA damage

Rym Sfaxi, Biswendu Biswas, Galina Boldina, Mandy Cadix, Nicolas Servant, Huimin Chen, Daniel Larson, Martin Dutertre, Caroline Robert, and Stephan Vagner
DOI: 10.15252/embj.2022112358

Corresponding author(s): Stephan Vagner (Stephan.Vagner@curie.fr)

Review Timeline:

Transfer from Review Commons:	12th Aug 22
Editorial Decision:	16th Aug 22
Revision Received:	23rd Dec 22
Editorial Decision:	20th Jan 23
Revision Received:	23rd Jan 23
Accepted:	25th Jan 23

Review COMMONS

Editor: Stefanie Boehm

Transaction Report: This manuscript was transferred to The EMBO JOURNAL following peer review at Review Commons.

Review #1

1. Evidence, reproducibility and clarity:

Evidence, reproducibility and clarity (Required)

Sfaxi et al., significantly extended a current knowledge of the mechanism and the function of co-transcriptional RNA cleavage (CoTC). First, the authors focused on TP53 genes to study the CoTC. They showed TP53 transcripts are cleaved independently of PCF11 and Pol II CTD Ser2 phosphorylation in the UV-treated cells, concluding RNA cleavage at polyadenylation site (PAS) of the TP53 gene occurs post-transcriptionally. This strongly suggests that TP53 gene transcription termination is regulated by CoTC. They also showed a biological importance of the CoTC on TP53 gene expression. Depletion of the potential TP53 CoTC genomic region impaired mRNA and protein levels of p53 and its target p21. This deregulated G1-S phase progression in cell cycle following UV treatment. Finally they extended these findings to other genes by the novel screening approach of the CoTC.

Minor comments:

1. P4, Second paragraph, Another model~; Two more papers need to be cited. "Dye and Proudfoot 2001 Cell" "West et al., 2008 Mol Cell"
2. Figure 3B; The authors should explain more about foci detected by probes A and B.
3. P10, Last line, strong decrease~; (Figures 4E and ~) -> (Figure 4E, right panels, and~)
4. Figure 5C; The author should show the entire image of the RNA-seq reads in the gene region, but not just in the windows described in Figure 5A. Also, TP53 and GAPDH genes need to be shown for the controls.

Referees cross-commenting

I totally agree with Reviewer #2.

2. Significance:

Significance (Required)

Overall this paper is well described and written. In my view, this will bring important information to the transcription termination field.

Note, I am not sure that the authors need to include the generality of the CoTC in Figures 5 and 6 since their RNA-seq analysis and its validation for biological functions are incomplete. Therefore I feel that focusing on the TP53 gene may enhance the impact of this paper.

3. How much time do you estimate the authors will need to complete the suggested revisions:

Estimated time to Complete Revisions (Required)

(Decision Recommendation)

Less than 1 month

4. Review Commons values the work of reviewers and encourages them to get credit for their work. Select 'Yes' below to register your reviewing activity at Publons; note that the content of your review will not be visible on Publons.

Reviewer Publons

Yes

Review #2

1. Evidence, reproducibility and clarity:

Evidence, reproducibility and clarity (Required)

DNA damage leads to transient inhibition of 3' end processing and a marked reduction of cellular Pcf11 levels, a component of the cleavage factor II (CF II). In previous work, St¬ephan Vagner and colleagues described that p53 mRNA escapes the 3' end processing inhibition through the actions of the DHX36 RNA-helicase and the heterogenous RNA binding protein hnRNP H/F. However, this previous work failed to identify a mechanism through which DHX36 and hnRNP H/F would mediate the

escape. In this work Stephane Vagner and his colleagues describe now how a sequence located 1200 nt downstream of the P53 poly(A) signal (PAS) is required to alleviate p53 mRNA from the general DNA-damage-dependent 3' end processing block. The presence of this element makes p53 mRNA 3' end processing independent from CTD-serine-2-phosphorylation and the CFII factor Pcf 11. As a high proportion of non-cleaved p53 pre-mRNA can be found in the nucleoplasm, the authors suggest that this element may be a co-transcriptional cleavage inducing site (CoTC). Deletion of this element renders p53 mRNA susceptible to DNA-damage-induced 3' end processing inhibition and leads to a p53 protein reduction after UV-exposure. Consequently, expression of down-stream targets of p53 is also reduced. The authors identify 9 more candidates for CoTC in other genes encoding for proteins required for the DNA-damage response.

Major points:

Although Figures 4 and S7 clearly show how long the RNA is that is retained on the chromatin, it is not clear, how long the RNA is that is released into the nucleoplasm. A Co-TC mechanism would pose that either nucleoplasmic CPSF-mediated cleavage and/or nucleoplasmic exonucleolytic degradation preceded nucleoplasmic polyadenylation. Neither of these points has been addressed by the current manuscript.
This could be addressed by transcript reverse transcription analysis with nuclear RNA, in the background of an exosome ko to see if this would allow stabilisation of the un-cleaved RNA and its detection in the nucleoplasmic fraction.

Minor points:

It would be good if the site of action of DHX36 and hnRNP H/F could be repeated and put into context with the Co-TC element.

Introduction; I feel the pause-type termination is probably better explained in either Cortazar et al 2019 or Gromak et al 2006.

There may be a typo towards the end of the second paragraph: vcxPAS. If this is not a typo, it would be helpful to have this explained better.

Figure 1A/B the conclusion: "These observations suggest that PCF11 might be dispensable for pre-mRNA 3' end processing following UV-induced DNA damage" is in contradiction to the general 3' end processing defect following DNA-damage that the authors cite. This statement is only understandable with the prior knowledge of

p53 mRNA being able to escape this inhibition. I feel it would be helpful to rephrase this.

Figure 1 E): shifting the entire axis up, is to my reading counterintuitive. I would show all graphs at the same scale.

Williamson et al (Svejstrup) 2017 showed through DRB-washed PRO-seq profiles that Pol II elongation speed is reduced for at least 8 hrs following UV-exposure, resulting in depletion from Pol II in gene bodies and concentration towards the gene beginnings (transcript start sites, TSS). It would be helpful to have an idea how the transcriptional profile looks on p53 at the time point of analysis - and at which time point after DNA-damage insult the 3' end processing inhibition starts. Such study could also form the beginning of a more in-depth analysis on how the CoTC is mediated.
Such an in-depth analysis should also probe the chromatin crosslinking of RNA Pol II, as well as 3' end factors at the regular PAS and downstream Co-TC element. The fact that the smFISH shows signal for the downstream probes of Rad53, suggests that Pol II regularly transcribes to these positions. Could there be another PAS-dependent termination signal in further downstrea areas?

Figure 5) the sequencing procedure should be explained better in the main text. From the text it is not clear, what sort of sequencing was performed.

Referees cross-commenting

@ Reviewer #1:

Generally agree with Reviewer 1.
2) Figure 3B : I agree that this experiment would benefit from better description. In fact, wouldnt one expect to be there signal in Figure 3D after UV, since cleavage is inhibited? Unless Williamson et al is taken into account, showing that transcription is generally slowed.

@ Reviewer #3:

Generally find all these suggestions are very valid and would increase the value of the manuscript.
I am not sure if I understand correctly/agree with two comments:

If understood correctly, this reviewer suggests that there is no Pcf11 under UV treatment conditions as suggested in Figure 1A. However, Figure 1B might show a

longer Western Blot exposure or have more material loaded, showing that there is some Pcf11 available for si-mediated knockdown.

Figure 5 Panel B. I would agree that this panel is not very well explained. My interpretation so far has been in quartiles (left top, less cleaved, less total; top right less cleaved, more total upon UV versus bottom left more cleaved, downregulated and bottom right more cleaved upreagulated upon UV). In which case a significant number of transcripts is no cleaved upon UV. To help with interpretation at least a longer legend could be added.

2. Significance:

Significance (Required)

This manuscript is overall convincing and adds more gravitas to the highly debated observation of co-transcriptional cleavage events. Although this study is by no means mechanistically exhaustive, it shows that the proposed mechanism may be true for the genes of DNA-damage repair factors that need to be "exempted" from the general DNA-damage-induced inhibition of 3' end cleavage. This opens up the exciting possibility that co-transcriptional elements can be used under specific, controlled environmental conditions. Although alternative explanations are possible to explain these pre-mRNA's release from the chromatin, 3' end processing at the regular poly(A) signal is for these RNAs clearly inhibited.

For a complete classification as Co-TC element however, additional experiments would be required. I am not adding these to the major or minor points, as these in my eyes would constitute a new story.
The original literature on CoTC (West et al 2004, Teixeira et al 2004), posed that a Co-TC event provides a 5'phospho entry site that could be used by a molecular torpedo (Xrn2). Part of the controversy about Co-TC cleavage is the question of how such a 5'phospho-end could chemically be generated by autocatalytic cleavage. To substantiate the claim that these elements are indeed Co-TC cleavage events either generated by auto-catalytic cleavage or another enzymatic function, the authors should test if they promote termination in this homologous, as well as a heterologous context.

3. How much time do you estimate the authors will need to complete the suggested revisions:

Estimated time to Complete Revisions (Required)

(Decision Recommendation)

Between 1 and 3 months

4. Review Commons values the work of reviewers and encourages them to get credit for their work. Select 'Yes' below to register your reviewing activity at Publons; note that the content of your review will not be visible on Publons.

Reviewer Publons

Yes

Review #3

1. Evidence, reproducibility and clarity:

Evidence, reproducibility and clarity (Required)

In this paper by the Vagner lab, co-transcriptional cleavage (CoTC) is discovered as a mechanism to ensure 3'-end processing of selected genes under conditions of global inhibition of 3'-end processing under DNA damage conditions.

While global inhibition of 3'-end processing acts as a fail-safe mechanism to ensure that mutations arising from DNA damage are not propagated (and instead repaired), the expression of repair genes must be maintained under these conditions. In an elegant serious of experiments, Sfaxi and coauthors have identified CoTC as a mechanism, which allows specific pre-mRNAs to escape 3'-end processing inhibition in response to UV-induced DNA damage.

This is an important discovery, the article reads well and most of the experiments are technically sound. That having said, I think the authors did not 'sell' this important finding very well. While they initially started their studies based on processing of the p53 pre-mRNA, they later performed RNAseq (Figure 5&6) to identify further genes that are regulated in a CoTC-dependent manner - analogous to the p53 pre-mRNA. In

doing so, the authors identify and validate further CoTC-dependent genes. Given the global RNAseq approach that the authors have undertaken but not yet fully exhausted, there are probably more genes hidden that are processed in a similar way.

I would recommend harvesting the hidden treasure to more comprehensively understand CoTC-dependent processing and its relation to DNA damage conditions. In my opinion, it would be important to perform

1. GO enrichment analyses of the 108 pre-mRNAs more effectively processed under UV and address the question whether DNA damage repair-related terms can be retrieved
2. study of further DNA-damaging conditions to address the question if similar patterns and genes can be retrieved under these conditions
3. complementary analysis of data derived from tumor genome databases whether mutations in the identified CoTC-sites are enriched (which one would expect)

Finally I wonder, whether a small scheme illustrating conventional versus CoTC processing could enhance access for a broader readership.

Minor Comments:

Page 4, 2nd paragraph, last sentence: what is "vcxPAS"?

Page 6, 2nd paragraph, the TBP RNA is not explained.

Page 6, 2nd paragraph and following paragraphs. The PCF11-depletion experiments to sort out conventional versus CoTC-dependence is not very well controlled: it is surprising to see that depletion of PCF11, which is already absent under UV (Fig. 1A), seems to modulate processing of TBP under this condition (Fig. 1E). In order to turn this into a bona fide positive control for the entire experimental set-up, it is relevant to show that there are residual PCF11-levels under UV that can be further downregulated by siRNAs under this condition (currently this is not supported by the WB data in Fig. 1B).

Page 8, 1st paragraph, last sentence: I find the reference to GAPDH and WDR13 as part of a figure that comes far below is a bit confusing

Page 9, last paragraph, page 10, 1st paragraph: What does the analysis of WDR12, GAPDH and TBP exactly control for?

Figure 3. Overall, I find the information shown in Fig. 3 somewhat confusing and wonder whether the quality can be improved (partial co-localization of spots, are the spots shown in D -UV an artifact? Etc.)

Figure 4. I find the composition of panels C and D not very intuitive (I would reorganize the data such that each panel shows the RNA and protein expression data for each candidate individually (panel C for p53 and panel D for p21, respectively)

Figure 5, panel B: The figure shows that the by far largest number of genes (>3000/3722) is not differentially regulated under UV compared to no-UV conditions. Does this question the commonly made statement that 3'-end processing is globally inhibited under UV quoted here (page 14, 2nd paragraph) and elsewhere?

Referees cross-commenting

@reviewer 1 & 3: I fully agree; the PCF11 depletion under UV-conditions is clearly visible. Thank you!

2. Significance:

Significance (Required)

This is an important study to better understand the function and target genes of CoTC-mediated 3'-end processing. It thereby extends earlier studies mainly adressing the underlying molecular mechanisms and rationalizes the function (and evolution) of this gene regulatory principle.

3. How much time do you estimate the authors will need to complete the suggested revisions:

Estimated time to Complete Revisions (Required)

(Decision Recommendation)

Between 1 and 3 months

4. Review Commons values the work of reviewers and encourages them to get credit for their work. Select 'Yes' below to register your reviewing activity at Publons; note

that the content of your review will not be visible on Publons.

Reviewer Publons

Yes

Revision Plan

Manuscript number: RC-2022-01508
Corresponding author(s): Stéphan VAGNER

1. General Statements

We would like to thank all three reviewers for their constructive suggestions. We were very excited to read that the reviewers considered the data well described and convincing and our findings representing an important information.
All new additions and changes to the manuscript, that have been or will be performed, are explained in our point-by-point response below.

2. Description of the planned revisions

Insert here a point-by-point reply that explains what revisions, additional experimentations and analyses are planned to address the points raised by the referees.

Reviewer #1 (Evidence, reproducibility and clarity (Required)):

Sfaxi et al., significantly extended a current knowledge of the mechanism and the function of co-transcriptional RNA cleavage (CoTC). First, the authors focused on TP53 genes to study the CoTC. They showed TP53 transcripts are cleaved independently of PCF11 and Pol II CTD Ser2 phosphorylation in the UV-treated cells, concluding RNA cleavage at polyadenylation site (PAS) of the TP53 gene occurs post-transcriptionally. This strongly suggests that TP53 gene transcription termination is regulated by CoTC. They also showed a biological importance of the CoTC on TP53 gene expression. Depletion of the potential TP53 CoTC genomic region impaired mRNA and protein levels of p53 and its target p21. This deregulated G1-S phase progression in cell cycle following UV treatment. Finally they extended these findings to other genes by the novel screening approach of the CoTC.

Minor comments:

2) Figure 3B; The authors should explain more about foci detected by probes A and B.

Reply (R): This will be done.

4) Figure 5C; The author should show the entire image of the RNA-seq reads in the gene region, but not just in the windows described in Figure 5A. Also, TP53 and GAPDH genes need to be shown for the controls.

R: This point will be addressed by showing RNA-seq for wider gene regions and for more control genes, as requested.

Reviewer #1 (Significance (Required)):

Overall this paper is well described and written. In my view, this will bring important information to the transcription termination field.

Reviewer #2 (Evidence, reproducibility and clarity (Required)):

DNA damage leads to transient inhibition of 3' end processing and a marked reduction of cellular Pcf11 levels, a component of the cleavage factor II (CF II). In previous work, Stéphan Vagner and colleagues described that p53 mRNA escapes the 3' end processing inhibition through the actions of the DHX36 RNA-helicase and the heterogenous RNA binding protein hnRNP H/F. However, this previous work failed to identify a mechanism through which DHX36 and hnRNP H/F would mediate the escape. In this work Stephane Vagner and his colleagues describe now how a sequence located 1200 nt downstream of the P53 poly(A) signal (PAS) is required to alleviate p53 mRNA from the general DNA-damage-dependent 3' end processing block. The presence of this element makes p53 mRNA 3' end processing independent from CTD-serine-2-phosphorylation and the CFII factor Pcf 11. As a high proportion of non-cleaved p53 pre-mRNA can be found in the nucleoplasm, the authors suggest that this element may be a co-transcriptional cleavage inducing site (CoTC). Deletion of this element renders p53 mRNA susceptible to DNA-damage-induced 3' end processing inhibition and leads to a p53 protein reduction after UV-exposure. Consequently, expression of down-stream targets of p53 is also reduced. The authors identify 9 more candidates for CoTC in other genes encoding for proteins required for the DNA-damage response.

Major points:
Although Figures 4 and S7 clearly show how long the RNA is that is retained on the chromatin, it is not clear, how long the RNA is that is released into the nucleoplasm. A Co-TC mechanism would pose that either nucleoplasmic CPSF-mediated cleavage and/or nucleoplasmic exonucleolytic degradation preceded nucleoplasmic polyadenylation. Neither of these points has been addressed by the current manuscript. This could be addressed by transcript reverse transcription analysis with nuclear RNA, in the background of an exosome ko to see if this would allow stabilisation of the un-cleaved RNA and its detection in the nucleoplasmic fraction.

R: We will perform this experiment.

Minor points:

Williamson et al (Svejstrup) 2017 showed through DRB-washed PRO-seq profiles that Pol II elongation speed is reduced for at least 8 hrs following UV-exposure, resulting in depletion from Pol II in gene bodies and concentration towards the gene beginnings (transcript start sites, TSS). It would be helpful to have an idea how the transcriptional profile looks on p53 at the time point of analysis - and at which time point after DNA-damage insult the 3' end processing inhibition starts. Such study could also form the beginning of a more in-depth analysis on how the CoTC is mediated.
Such an in-depth analysis should also probe the chromatin crosslinking of RNA Pol II, as well as 3' end factors at the regular PAS and downstream Co-TC element. The fact that the smFISH shows signal for the downstream probes of Rad53, suggests that Pol II regularly transcribes to these positions. Could there be another PAS-dependent termination signal in further downstrea areas?

R: These aspects will be discussed in the discussion section. We think, together with this reviewer, that this discussion will introduce interesting perspectives for future studies.

Reviewer #2 (Significance (Required)):

This manuscript is overall convincing and adds more gravitas to the highly debated observation of co-transcriptional cleavage events. Although this study is by no means mechanistically exhaustive, it shows that the proposed mechanism may be true for the genes of DNA-damage repair factors that need to be "exempted" from the general DNA-damage-induced inhibition of 3' end cleavage. This opens up the exciting possibility that co-transcriptional elements can be used under specific, controlled environmental conditions. Although alternative explanations are possible to explain these pre-mRNA's release from the chromatin, 3' end processing at the regular poly(A) signal is for these RNAs clearly inhibited.

For a complete classification as Co-TC element however, additional experiments would be required. I am not adding these to the major or minor points, as these in my eyes would constitute a new story.
The original literature on CoTC (West et al 2004, Teixeira et al 2004), posed that a Co-TC event provides a 5'phospho entry site that could be used by a molecular torpedo (Xrn2). Part of the controversy about Co-TC cleavage is the question of how such a 5'phospho-end could chemically be generated by autocatalytic cleavage. To substantiate the claim that these elements are indeed Co-TC cleavage events either generated by auto-catalytic cleavage or another enzymatic function, the authors should test if they promote termination in this homologous, as well as a heterologous context.

R: This interesting point will be added in the discussion.

Reviewer #3 (Evidence, reproducibility and clarity (Required)):

In this paper by the Vagner lab, co-transcriptional cleavage (CoTC) is discovered as a mechanism to ensure 3'-end processing of selected genes under conditions of global inhibition of 3'-end processing under DNA damage conditions.

While global inhibition of 3'-end processing acts as a fail-safe mechanism to ensure that mutations arising from DNA damage are not propagated (and instead repaired), the expression of repair genes must be maintained under these conditions. In an elegant serious of experiments, Sfaxi and coauthors have identified CoTC as a mechanism, which allows specific pre-mRNAs to escape 3'-end processing inhibition in response to UV-induced DNA damage.

This is an important discovery, the article reads well and most of the experiments are technically sound. That having said, I think the authors did not 'sell' this important finding very well. While they initially started their studies based on processing of the p53 pre-mRNA, they later performed RNAseq (Figure 5&6) to identify further genes that are regulated in a CoTC-dependent manner - analogous to the p53 pre-mRNA. In doing so, the authors identify and validate further CoTC-dependent genes. Given the global RNAseq approach that the authors have undertaken but not yet fully exhausted, there are probably more genes hidden that are processed in a similar way.

In my opinion, it would be important to perform

(2) study of further DNA-damaging conditions to address the question if similar patterns and genes can be retrieved under these conditions

R: We will test another DNA damaging condition, as it was done for UV in Fig. 4A.

Finally I wonder, whether a small scheme illustrating conventional versus CoTC processing could enhance access for a broader readership.

R: We will add a schematic illustration.

Minor comments

Figure 3. Overall, I find the information shown in Fig. 3 somewhat confusing and wonder whether the quality can be improved (partial co-localization of spots, are the spots shown in D - UV an artifact? Etc.)

R: This will be addressed.

Figure 5, panel B: The figure shows that the by far largest number of genes (>3000/3722) is not differentially regulated under UV compared to no-UV conditions. Does this question the commonly made statement that 3'-end processing is globally inhibited under UV quoted here (page 14, 2nd paragraph) and elsewhere?

R: The commonly made statement that 3'-end processing is globally inhibited under UV is based on the strong decrease of poly(A) site cleavage activity that is observed following UV irradiation, in experiments using cellular extracts and poly(A) site reporters. As noted by the reviewer, in Fig. 5B, we detect an increase in downstream/upstream reads ratio (uncleaved/total RNA) for only 378 genes. In other words, we do not see a massive increase in transcriptional readthrough beyond the 3'-end of most genes. We will discuss this point in the discussion. First, it should be kept in mind that our RNA-seq analysis is only analyzing 4208 genes, and only their last poly(A) site (not the many alternative ones). Second, there are potential limitations in our RNA-seq analysis, such as the size of windows (500 nucleotides) upstream/downstream of the poly(A) site, and the analysis of nuclear RNA rather than chromatin-bound or nascent RNA (indeed, decreased cleavage at poly(A) site is expected to decrease the release of transcript from chromatin and therefore its solubility). Third, for some genes, decreased cleavage at poly(A) site upon UV might be compensated by either increased degradation of uncleaved RNA or increased splicing to a downstream exon (either an unannotated distal alternative last exon or an exon in a downstream gene). Fourth, the extent of cleavage inhibition may depend on UV dose and time. Nevertheless, the main point we are making from our RNA-seq analysis is that a specific subset of genes (108 out of 4208 genes) exhibits an increase in poly(A) site cleavage efficiency upon UV irradiation.

CROSS-CONSULTATION COMMENTS
@reviewer 1 & 3: I fully agree; the PCF11 depletion under UV-conditions is clearly visible. Thank you!

Reviewer #3 (Significance (Required)):

This is an important study to better understand the function and target genes of CoTC-mediated 3'-end processing. It thereby extends earlier studies mainly adressing the underlying molecular mechanisms and rationalizes the function (and evolution) of this gene regulatory principle.

3. Description of the revisions that have already been incorporated in the transferred manuscript

Please insert a point-by-point reply describing the revisions that were already carried out and included in the transferred manuscript. If no revisions have been carried out yet, please leave this section empty.

Reviewer #1 (Evidence, reproducibility and clarity (Required)):

Minor comments:
1) P4, Second paragraph, Another model~; Two more papers need to be cited. "Dye and Proudfoot 2001 Cell" "West et al., 2008 Mol Cell"

Reply (R): We fully agree and we have now added these 2 references. We are sorry for having forgotten these 2 references.

3) P10, Last line, strong decrease~; (Figures 4E and ~) -> (Figure 4E, right panels, and~)

R: We have removed the word "*strong*" that was indeed not necessary.

CROSS-CONSULTATION COMMENTS
I totally agree with Reviewer #2.

Reviewer #1 (Significance (Required)):

Overall this paper is well described and written. In my view, this will bring important information to the transcription termination field.

Note, I am not sure that the authors need to include the generality of the CoTC in Figures 5 and 6 since their RNA-seq analysis and its validation for biological functions are incomplete. Therefore I feel that focusing on the TP53 gene may enhance the impact of this paper.

R: We think that keeping figures 5 and 6 will help to enhance the impact of the paper. It indeed contributes to provide a list of CoTC candidate genes including several ones that are in the p53 pathway.
We have also now performed and added GO analysis of the CoTC-containing gene candidates resulting from the RNA-seq analysis (new Supplementary Figure 9) that shows an enrichment of genes involved in the inhibition of apoptosis and metabolism of a genotoxic stress inducing agent, doxorubicin.

Reviewer #2

Minor points:
It would be good if the site of action of DHX36 and hnRNP H/F could be repeated and put into context with the Co-TC element.

R: We have added new supplementary figure (N°2) and the following text in the manuscript: “We have previously shown that the two RBPs hnRNP H/F (Decorsière et al., 2011) and the RNA helicase DHX36 (Newman et al., 2017) are involved in the regulation of *p53* pre-mRNA 3’-end processing following UV-induced DNA damage. Consistent with *p53* pre-mRNA 3’-end processing mostly occurring in the nucleoplasm, the increased uncleaved/total ratio of *p53* pre-mRNA following hnRNP H/F depletion was significantly higher in the nucleoplasm than in the chromatin (Supplementary Figure 2). “

Introduction; I feel the pause-type termination is probably better explained in either Cortazar et al 2019 or Gromak et al 2006.

R: We have now added the references Cortazak et al. Mol Cell 2019 and Gromak et al. Mol Cell Biol 2016.

There may be a typo towards the end of the second paragraph: vcxPAS. If this is not a typo, it would be helpful to have this explained better.

R: We have now corrected it.

Figure 1A/B the conclusion: "These observations suggest that PCF11 might be dispensable for pre-mRNA 3' end processing following UV-induced DNA damage" is in contradiction to the general 3' end processing defect following DNA-damage that the authors cite. This statement is only understandable with the prior knowledge of p53 mRNA being able to escape this inhibition. I feel it would be helpful to rephrase this.

R: We thank the referee for indicating this mistake. We have corrected the sentence: “These observations suggest that PCF11 might be dispensable for *p53* pre-mRNA 3' end processing following UV-induced DNA damage.”

Figure 1 E): shifting the entire axis up, is to my reading counterintuitive. I would show all graphs at the same scale.

R: We think that the axis should be adapted to each graph in order to appreciate the differences observed between different conditions of each experiment.

Figure 5) the sequencing procedure should be explained better in the main text. From the text it is not clear, what sort of sequencing was performed.

R: We have now clarified the RNA-seq procedure in the main text (page 11): “Toward this aim, we first developed a strategy to analyze in a high-throughput manner the efficiency of pre-mRNA 3’-end cleavage by RNA sequencing (RNA-Seq) on nuclear RNA depleted of ribosomal RNA. This strategy is based on the evaluation of the number of reads located in 500 nt-long windows either upstream (total RNA) or downstream of the PAS (PAS-uncleaved RNA; Figure 4A).” More detailed information on the RNA-seq procedure is provided in the Materials and Methods section.

CROSS-CONSULTATION COMMENTS
@ Reviewer #1:
Generally agree with Reviewer 1.
2) Figure 3B : I agree that this experiment would benefit from better description. In fact, wouldnt one expect to be there signal in Figure 3D after UV, since cleavage is inhibited? Unless Williamson et al is taken into account, showing that transcription is generally slowed.

R: This point will be added in the discussion.

@ Reviewer #3:
Generally find all these suggestions are very valid and would increase the value of the manuscript.
I am not sure if I understand correctly/agree with two comments:
If understood correctly, this reviewer suggests that there is no Pcf11 under UV treatment conditions as suggested in Figure 1A. However, Figure 1B might show a longer Western Blot exposure or have more material loaded, showing that there is some Pcf11 available for si-mediated knockdown.

R: We repeated the western blotting of PCF11 (Fig. 1A) with a different exposition time that shows the decreased abundance of PCF11 in UV-treated conditions (new panel in Fig.1A)

Figure 5 Panel B. I would agree that this panel is not very well explained. My interpretation so far has been in quartiles (left top, less cleaved, less total; top right less cleaved, more total upon UV versus bottom left more cleaved, downregulated and bottom right more cleaved upreagulated upon UV). In which case a significant number of transcripts is no cleaved upon UV. To help with interpretation at least a longer legend could be added.

R: We have improved the legend of Fig. 5B, as follows: "(B) RNA-seq plot representing, for each gene, the log2 fold change (comparing UV-irradiated to non-irradiated cells) of the number of reads downstream of the poly(A) site (uncleaved RNA, *y* axis), and the log2 FC of the number of reads upstream of the poly(A) site (total RNA, *x* axis). Regulation events are considered significant if p-value is below 0.05. The 108 genes at bottom right have a decreased uncleaved/total RNA ratio, meaning that PAS cleavage is increased upon UV irradiation. In contrast, the 378 genes at top left have an increased uncleaved/total RNA ratio, meaning that PAS cleavage is decreased upon UV irradiation."

Reviewer #3

I would recommend harvesting the hidden treasure to more comprehensively understand CoTC-dependent processing and its relation to DNA damage conditions. In my opinion, it would be important to perform

(1) GO enrichment analyses of the 108 pre-mRNAs more effectively processed under UV and address the question whether DNA damage repair-related terms can be retrieved

R: We have now performed and added GO analysis of the CoTC-containing gene candidates resulting from the RNA-seq analysis (new Supplementary Figure 9) that shows an enrichment of

genes involved in the inhibition of apoptosis and metabolism of a genotoxic stress inducing agent, doxorubicin.

Minor Comments:

Page 4, 2nd paragraph, last sentence: what is "vcxPAS"?

R: Sorry for this typographic error. We have corrected it.

Page 6, 2nd paragraph, the TBP RNA is not explained.

R: We indicated in the text that TBP is used as a negative control to test the PCF11-dependence of *p53* pre-mRNA 3'end processing.

Page 6, 2nd paragraph and following paragraphs. The PCF11-depletion experiments to sort out conventional versus CoTC-dependence is not very well controlled: it is surprising to see that depletion of PCF11, which is already absent under UV (Fig. 1A), seems to modulate processing of TBP under this condition (Fig. 1E). In order to turn this into a bona fide positive control for the entire experimental set-up, it is relevant to show that there are residual PCF11-levels under UV that can be further downregulated by siRNAs under this condition (currently this is not supported by the WB data in Fig. 1B).

R: We repeated the western blotting of PCF11 (Figure 1A) with a different exposition time that shows the decreased abundance of PCF11 in UV-treated conditions (new panel in Fig. 1A)

Page 8, 1st paragraph, last sentence: I find the reference to GAPDH and WDR13 as part of a figure that comes far below is a bit confusing

R: We agree and we removed this reference to avoid confusion.

Page 9, last paragraph, page 10, 1st paragraph: What does the analysis of WDR12, GAPDH and TBP exactly control for?

R: WDR13 is a CoTC regulated candidate (Nojima et al 2013), hence serves as positive control whereas GAPDH (Nojima et al 2013) and TBP are not regulated by CoTC as their 3' end cleavage efficiency decreases upon UV treatment, hence they serve as negative controls. In p53-CoTC deleted cells they serve to control the specificity of the effect of the deletion on the p53 3' end cleavage efficiency and not for the control candidates.

4. Description of analyses that authors prefer not to carry out

Please include a point-by-point response explaining why some of the requested data or additional analyses might not be necessary or cannot be provided within the scope of a revision. This can be due to time or resource limitations or in case of disagreement about the necessity of such additional data given the scope of the study. Please leave empty if not applicable.

Reviewer #3

(3) complementary analysis of data derived from tumor genome databases whether mutations in the identified CoTC-sites are enriched (which one would expect)

R: This is a very important aspect that will provide a starting point to explore the importance of the CoTC sequences in cancer initiation or progression. We however believe that it is out of the scope of this manuscript that investigates the role of the CoTC in the response to genotoxic stress.

Minor points

Figure 4. I find the composition of panels C and D not very intuitive (I would reorganize the data such that each panel shows the RNA and protein expression data for each candidate individually (panel C for p53 and panel D for p21, respectively)

R: Our representation illustrates the changes in RNA expression first and then we explain how this is coherent with protein expression levels by western blotting.

1st Editorial Decision 16th Aug 2022

Thank you for transferring your Review Commons manuscript to The EMBO Journal, as well as your response to the referee comments. In light of the referees' positive comments and your revision plan, we would like to invite you to prepare and submit a revised manuscript to The EMBO Journal.

As outlined in your revision plan, it will be important to address the reviewer's concerns experimentally or by revising the manuscript and to provide a careful response to each of their comments. In particular, referee #2's major point as well as referee #3' major point 2 should be addressed experimentally and all necessary controls and descriptions of experimental details must be added. In addition, the effect of PCF11 depletion before and upon UV treatment must be clarified. Here, please add further information to the Figure 1 legends and specify that these are different sub-complexes in the same cell line, in addition, it is not obvious why the blots for GAPDH and PCF11 in panel A CFIIm and B differ (single vs. double bands). Please also carefully respond to all other referee concerns in a single point-by-point response to all of the initial comments from Review Commons. In addition, please remember that the manuscript should fulfill all EMBO Journal formatting requirements at the time-point of re-submission and in particular ensure that all figures adhere to the journal's requirements (for example Figure 3- please add scale bars, statistics on cells analyzed, acquisition settings, etc).

Uncoupling from transcription protects polyadenylation site cleavage from inhibition by DNA damage
Sfaxi, Biswas et al.

We would like to thank all three reviewers for their constructive suggestions. We were very excited to read that the reviewers considered the data well described and convincing and our findings representing an important information.
All new additions and changes to the manuscript are explained in our point-by-point response below.

Reviewer #1 (Evidence, reproducibility and clarity (Required)):

Sfaxi et al., significantly extended a current knowledge of the mechanism and the function of co-transcriptional RNA cleavage (CoTC). First, the authors focused on TP53 genes to study the CoTC. They showed TP53 transcripts are cleaved independently of PCF11 and Pol II CTD Ser2 phosphorylation in the UV-treated cells, concluding RNA cleavage at polyadenylation site (PAS) of the TP53 gene occurs post-transcriptionally. This strongly suggests that TP53 gene transcription termination is regulated by CoTC. They also showed a biological importance of the CoTC on TP53 gene expression. Depletion of the potential TP53 CoTC genomic region impaired mRNA and protein levels of p53 and its target p21. This deregulated G1-S phase progression in cell cycle following UV treatment. Finally they extended these findings to other genes by the novel screening approach of the CoTC.

Minor comments:
1) P4, Second paragraph, Another model~; Two more papers need to be cited. "Dye and Proudfoot 2001 Cell" "West et al., 2008 Mol Cell"

Reply (R): We fully agree, and we have now added these 2 references.

2) Figure 3B; The authors should explain more about foci detected by probes A and B.

R: In response to this comment, we included in the text (and in Fig. S2) the quantification of the spots / nucleus for a CoTC transcript (p53) and a control (GAPDH), clearly showing the difference in abundance. We further added a sentence to the manuscript in regard to panel D.

3) P10, Last line, strong decrease~; (Figures 4E and ~) -> (Figure 4E, right panels, and~)

R: We have removed the word “*strong*” that was indeed not necessary.

4) Figure 5C; The author should show the entire image of the RNA-seq reads in the gene region, but not just in the windows described in Figure 5A. Also, TP53 and GAPDH genes need to be shown for the controls.

R: We agree that the former representation was confusing since no reads were shown for most of the gene region initially presented in the figure. In the new Figure 5, we have now shortened the gene region scheme to only show the sequencing reads that were analyzed (*i.e.* in a window of 500nts upstream and downstream of the PAS).

CROSS-CONSULTATION COMMENTS
I totally agree with Reviewer #2.

Reviewer #1 (Significance (Required)):

Overall this paper is well described and written. In my view, this will bring important

information to the transcription termination field.

Note, I am not sure that the authors need to include the generality of the CoTC in Figures 5 and 6 since their RNA-seq analysis and its validation for biological functions are incomplete. Therefore I feel that focusing on the TP53 gene may enhance the impact of this paper.

R: We think that keeping figures 5 and 6 will help to enhance the impact of the paper. It indeed contributes to provide a list of candidates CoTC genes, including several ones that are in the p53 pathway.
Also, in response to reviewer #3, we have also now performed and added GO analysis of the CoTC-containing gene candidates resulting from the RNA-seq analysis (new Figure EV2) that shows an enrichment of genes involved in the inhibition of apoptosis and metabolism of a genotoxic stress inducing agent, doxorubicin.

Reviewer #2 (Evidence, reproducibility and clarity (Required)):

DNA damage leads to transient inhibition of 3' end processing and a marked reduction of cellular Pcf11 levels, a component of the cleavage factor II (CF II). In previous work, Stéphan Vagner and colleagues described that p53 mRNA escapes the 3' end processing inhibition through the actions of the DHX36 RNA-helicase and the heterogenous RNA binding protein hnRNP H/F. However, this previous work failed to identify a mechanism through which DHX36 and hnRNP H/F would mediate the escape. In this work Stephane Vagner and his colleagues describe now how a sequence located 1200 nt downstream of the P53 poly(A) signal (PAS) is required to alleviate p53 mRNA from the general DNA-damage-dependent 3' end processing block. The presence of this element makes p53 mRNA 3' end processing independent from CTD-serine-2-phosphorylation and the CFII factor Pcf 11. As a high proportion of non-cleaved p53 pre-mRNA can be found in the nucleoplasm, the authors suggest that this element may be a co-transcriptional cleavage inducing site (CoTC). Deletion of this element renders p53 mRNA susceptible to DNA-damage-induced 3' end processing inhibition and leads to a p53 protein reduction after UV-exposure. Consequently, expression of down-stream targets of p53 is also reduced. The authors identify 9 more candidates for CoTC in other genes encoding for proteins required for the DNA-damage response.

Major points:
Although Figures 4 and S7 clearly show how long the RNA is that is retained on the chromatin, it is not clear, how long the RNA is that is released into the nucleoplasm. A Co-TC mechanism would pose that either nucleoplasmic CPSF-mediated cleavage and/or nucleoplasmic exonucleolytic degradation preceded nucleoplasmic polyadenylation. Neither of these points has been addressed by the current manuscript. This could be addressed by transcript reverse transcription analysis with nuclear RNA, in the background of an exosome ko to see if this would allow stabilisation of the un-cleaved RNA and its detection in the nucleoplasmic fraction.

R: We have now performed the requested experiment. The results presented in the new Fig 2F show that the CoTC cleaved *p53* RNA can be detected in the nucleoplasm.
We have added the following sentences in the ms:
"*In addition, nucleoplasmic RNA was reverse-transcribed using random primers and the obtained cDNA was amplified by PCR as in Fig 2C. The bands obtained show that the CoTC-cleaved RNA can be detected in the nucleoplasm (Fig 2F). Altogether, these data indicate that the p53 pre-mRNAs cleaved in the vicinity of the AT-rich region (i) do not contain a poly(A) tail, (ii) are generated through a CoTC-type event in an UV-induced manner, and (iii) can be found in the nucleoplasm before PAS cleavage.*"

Minor points:
It would be good if the site of action of DHX36 and hnRNP H/F could be repeated and put into context with the Co-TC element.

R: We have added a new appendix figure (S3) and the following text in the manuscript: "*Of note, we have previously shown that hnRNP H/F (Decorsière et al, 2011) and DHX36 (Newman et al, 2017) are involved in the regulation of p53 pre-mRNA 3'-end processing following UV-induced DNA damage. Consistent with p53 pre-mRNA 3'-end processing mostly occurring in the nucleoplasm, the increased uncleaved/total ratio of p53 pre-mRNA following depletion of DHX36 or hnRNP H/F was significantly higher in the nucleoplasm than in the chromatin (Appendix Fig S3).*"

Introduction; I feel the pause-type termination is probably better explained in either Cortazar et al 2019 or Gromak et al 2006.

R: We have now added the references Cortazak et al. Mol Cell 2019 and Gromak et al. Mol Cell Biol 2016.

There may be a typo towards the end of the second paragraph: vcxPAS. If this is not a typo, it would be helpful to have this explained better.

R: We have now corrected it.

Figure 1A/B the conclusion: "These observations suggest that PCF11 might be dispensable for pre-mRNA 3' end processing following UV-induced DNA damage" is in contradiction to the general 3' end processing defect following DNA-damage that the authors cite. This statement is only understandable with the prior knowledge of p53 mRNA being able to escape this inhibition. I feel it would be helpful to rephrase this.

R: We thank the referee for indicating this mistake. We have corrected the sentence: "*These observations suggest that PCF11 might be dispensable for p53 pre-mRNA 3' end processing following UV-induced DNA damage*."

Figure 1 E): shifting the entire axis up, is to my reading counterintuitive. I would show all graphs at the same scale.

R: We think that the axis should be adapted to each graph in order to appreciate the differences observed between different conditions of each experiment.

Williamson et al (Svejstrup) 2017 showed through DRB-washed PRO-seq profiles that Pol II elongation speed is reduced for at least 8 hrs following UV-exposure, resulting in depletion from Pol II in gene bodies and concentration towards the gene beginnings (transcript start sites, TSS). It would be helpful to have an idea how the transcriptional profile looks on p53 at the time point of analysis - and at which time point after DNA-damage insult the 3' end processing inhibition starts. Such study could also form the beginning of a more in-depth analysis on how the CoTC is mediated.
Such an in-depth analysis should also probe the chromatin crosslinking of RNA Pol II, as well as 3' end factors at the regular PAS and downstream Co-TC element. The fact that the smFISH shows signal for the downstream probes of Rad53, suggests that Pol II regularly transcribes to these positions. Could there be another PAS-dependent termination signal in further downstrea areas?

R: As mentioned by the reviewer, the fact that, using smFISH, *p53* pre-mRNA regions downstream of the PAS (Probe B, Fig. 3) were detected following UV exposure indicates that transcription occurs in PAS-downstream regions, even in UV-conditions. Of note, the

experiments were performed 16h after UV exposure, and not 8h after UV exposure as in Williamson et al.
The fact that the *p53* pre-mRNA cannot be reverse-transcribed in the PAS-downstream region (probes R1 to R6) when using an oligo dT for reverse transcription (Fig. 2E) indicates that there is no downstream alternative PAS.

Figure 5) the sequencing procedure should be explained better in the main text. From the text it is not clear, what sort of sequencing was performed.

R: We have now clarified the RNA-seq procedure in the main text. See response to Reviewer #1.

CROSS-CONSULTATION COMMENTS
@ Reviewer #1:
Generally agree with Reviewer 1.
2) Figure 3B : I agree that this experiment would benefit from better description. In fact, wouldnt one expect to be there signal in Figure 3D after UV, since cleavage is inhibited? Unless Williamson et al is taken into account, showing that transcription is generally slowed.

R: We extensively modified the text and we included in the text (and in Fig. S2) the quantification of the spots / nucleus for a CoTC transcript (p53) and a control (GAPDH), clearly showing the difference in abundance.
We further added a sentence to the manuscript in regard to panel D: "*It is possible that these GAPDH spots in the nucleus reflect transcription past the termination sequence, which is now understood to be widespread (Vilborg et al, 2015).*"

@ Reviewer #3:
Generally find all these suggestions are very valid and would increase the value of the manuscript.
I am not sure if I understand correctly/agree with two comments:
If understood correctly, this reviewer suggests that there is no Pcf11 under UV treatment conditions as suggested in Figure 1A. However, Figure 1B might show a longer Western Blot exposure or have more material loaded, showing that there is some Pcf11 available for si-mediated knockdown.

R: We repeated the western blotting of PCF11 (Fig. 1A) with a different exposure time; it shows a decreased abundance of PCF11 in UV-treated conditions (new panel in Fig.1A)

Figure 5 Panel B. I would agree that this panel is not very well explained. My interpretation so far has been in quartiles (left top, less cleaved, less total; top right less cleaved, more total upon UV versus bottom left more cleaved, downregulated and bottom right more cleaved upreagulated upon UV). In which case a significant number of transcripts is no cleaved upon UV. To help with interpretation at least a longer legend could be added.

R: We have improved the legend of Fig. 5B, as follows: *"(B) RNA-seq plot representing, for each gene, the log2 fold change (comparing UV-irradiated to non-irradiated cells) of the number of reads downstream of the poly(A) site (uncleaved RNA, y axis), and the log2 FC of the number of reads upstream of the poly(A) site (total RNA, x axis). Regulation events are considered significant if p-value is below 0.05. The 108 genes at bottom right have a decreased uncleaved/total RNA ratio, meaning that PAS cleavage is increased upon UV irradiation. In contrast, the 378 genes at top left have an increased uncleaved/total RNA ratio, meaning that PAS cleavage is decreased upon UV irradiation.*"

Reviewer #2 (Significance (Required)):

This manuscript is overall convincing and adds more gravitas to the highly debated observation of co-transcriptional cleavage events. Although this study is by no means mechanistically exhaustive, it shows that the proposed mechanism may be true for the genes of DNA-damage repair factors that need to be "exempted" from the general DNA-damage-induced inhibition of 3' end cleavage. This opens up the exciting possibility that co-transcriptional elements can be used under specific, controlled environmental conditions. Although alternative explanations are possible to explain these pre-mRNA's release from the chromatin, 3' end processing at the regular poly(A) signal is for these RNAs clearly inhibited.

For a complete classification as Co-TC element however, additional experiments would be required. I am not adding these to the major or minor points, as these in my eyes would constitute a new story.
The original literature on CoTC (West et al 2004, Teixeira et al 2004), posed that a Co-TC event provides a 5'phospho entry site that could be used by a molecular torpedo (Xrn2). Part of the controversy about Co-TC cleavage is the question of how such a 5'phospho-end could chemically be generated by autocatalytic cleavage. To substantiate the claim that these elements are indeed Co-TC cleavage events either generated by auto-catalytic cleavage or another enzymatic function, the authors should test if they promote termination in this homologous, as well as a heterologous context.

R: We believe that clarifying this controversy is out of the scope of this ms. This would require an extensive set of experiments. This is however an interesting point that could be addressed with all the candidate CoTC genes we identified in this study.

Reviewer #3 (Evidence, reproducibility and clarity (Required)):

In this paper by the Vagner lab, co-transcriptional cleavage (CoTC) is discovered as a mechanism to ensure 3'-end processing of selected genes under conditions of global inhibition of 3'-end processing under DNA damage conditions.

While global inhibition of 3'-end processing acts as a fail-safe mechanism to ensure that mutations arising from DNA damage are not propagated (and instead repaired), the expression of repair genes must be maintained under these conditions. In an elegant serious of experiments, Sfaxi and coauthors have identified CoTC as a mechanism, which allows specific pre-mRNAs to escape 3'-end processing inhibition in response to UV-induced DNA damage.

This is an important discovery, the article reads well and most of the experiments are technically sound. That having said, I think the authors did not 'sell' this important finding very well. While they initially started their studies based on processing of the p53 pre-mRNA, they later performed RNAseq (Figure 5&6) to identify further genes that are regulated in a CoTC-dependent manner - analogous to the p53 pre-mRNA. In doing so, the authors identify and validate further CoTC-dependent genes. Given the global RNAseq approach that the authors have undertaken but not yet fully exhausted, there are probably more genes hidden that are processed in a similar way.

I would recommend harvesting the hidden treasure to more comprehensively understand CoTC-dependent processing and its relation to DNA damage conditions. In my opinion, it would be important to perform

(1) GO enrichment analyses of the 108 pre-mRNAs more effectively processed under UV and address the question whether DNA damage repair-related terms can be retrieved

R: We have now performed and added GO analysis of the CoTC-containing gene candidates resulting from the RNA-seq analysis (new Figure EV2) that shows an enrichment of genes

involved in the inhibition of apoptosis and metabolism of a genotoxic stress inducing agent, doxorubicin.

(2) study of further DNA-damaging conditions to address the question if similar patterns and genes can be retrieved under these conditions

R: We have tested doxorubicin treatment as a new DNA damaging condition since (i) genes related to doxorubicin metabolism were found enriched among the UV-resistant pre-mRNAs (Fig. EV2) and (ii) *p53* pre-mRNA 3'-end processing is maintained upon doxorubicin treatment (Decorsière *et al*, 2011). As presented in the new Fig EV3 and Expanded Fig S11, we found an association between maintained 3'-end processing following doxorubicin treatment, and the presence of a CoTC element in *p53* and all 15 candidate pre-mRNAs that are resistant to UV exposure.

(3) complementary analysis of data derived from tumor genome databases whether mutations in the identified CoTC-sites are enriched (which one would expect)

R: This is a very important aspect that will provide a starting point to explore the importance of the CoTC sequences in cancer initiation or progression. We however believe that it is out of the scope of this manuscript that investigates the role of the CoTC in the response to genotoxic stress.

Finally I wonder, whether a small scheme illustrating conventional versus CoTC processing could enhance access for a broader readership.

R: We have added a scheme in the new Appendix Fig S12.

Minor Comments:

Page 4, 2nd paragraph, last sentence: what is "vcxPAS"?

R: Sorry for this typographic error. We have corrected it.

Page 6, 2nd paragraph, the TBP RNA is not explained.

R: We indicated in the text that TBP is used as a negative control to test the PCF11-dependence of *p53* pre-mRNA 3'end processing.

Page 6, 2nd paragraph and following paragraphs. The PCF11-depletion experiments to sort out conventional versus CoTC-dependence is not very well controlled: it is surprising to see that depletion of PCF11, which is already absent under UV (Fig. 1A), seems to modulate processing of TBP under this condition (Fig. 1E). In order to turn this into a bona fide positive control for the entire experimental set-up, it is relevant to show that there are residual PCF11-levels under UV that can be further downregulated by siRNAs under this condition (currently this is not supported by the WB data in Fig. 1B).

R: We repeated the western blotting of PCF11 (Figure 1A) with a different exposition time that shows the decreased abundance of PCF11 in UV-treated conditions (new panel in Fig. 1A)

Page 8, 1st paragraph, last sentence: I find the reference to GAPDH and WDR13 as part of a figure that comes far below is a bit confusing

R: We agree, and we removed this reference to avoid confusion.

Page 9, last paragraph, page 10, 1st paragraph: What does the analysis of WDR12, GAPDH and TBP exactly control for?

R: WDR13 is a CoTC regulated candidate (Nojima et al 2013), hence serves as positive control whereas GAPDH (Nojima et al 2013) and TBP are not regulated by CoTC as their 3' end cleavage efficiency decreases upon UV treatment, hence they serve as negative controls. In p53-CoTC deleted cells they serve to control the specificity of the effect of the deletion on the p53 3' end cleavage efficiency and not for the control candidates.

Figure 3. Overall, I find the information shown in Fig. 3 somewhat confusing and wonder whether the quality can be improved (partial co-localization of spots, are the spots shown in D -UV an artifact? Etc.)

R: We extensively modified the text and we included in the text (and in Fig. S2) the quantification of the spots / nucleus for a CoTC transcript (p53) and a control (GAPDH), clearly showing the difference in abundance.
We further added a sentence to the manuscript in regard to panel D: "*It is possible that these GAPDH spots in the nucleus reflect transcription past the termination sequence, which is now understood to be widespread (Vilborg et al, 2015).*"

Figure 4. I find the composition of panels C and D not very intuitive (I would reorganize the data such that each panel shows the RNA and protein expression data for each candidate individually (panel C for p53 and panel D for p21, respectively)

R: Our representation illustrates the changes in RNA expression first and then we explain how this is coherent with protein expression levels by western blotting.

Figure 5, panel B: The figure shows that the by far largest number of genes (>3000/3722) is not differentially regulated under UV compared to no-UV conditions. Does this question the commonly made statement that 3'-end processing is globally inhibited under UV quoted here (page 14, 2nd paragraph) and elsewhere?

R: The commonly made statement that 3'-end processing is globally inhibited under UV is based on the strong decrease of poly(A) site cleavage activity that is observed following UV irradiation, in experiments using cellular extracts and poly(A) site reporters. As noted by the reviewer, in Fig. 5B, we detect an increase in downstream/upstream reads ratio (uncleaved/total RNA) for only 378 genes. In other words, we do not see a massive increase in transcriptional readthrough beyond the 3'-end of most genes. We will discuss this point in the discussion. First, it should be kept in mind that our RNA-seq analysis is only analyzing 4208 genes, and only their last poly(A) site (not the many alternative ones). Second, there are potential limitations in our RNA-seq analysis, such as the size of windows (500 nucleotides) upstream/downstream of the poly(A) site, and the analysis of nuclear RNA rather than chromatin-bound or nascent RNA (indeed, decreased cleavage at poly(A) site is expected to decrease the release of transcript from chromatin and therefore its solubility). Third, for some genes, decreased cleavage at poly(A) site upon UV might be compensated by either increased degradation of uncleaved RNA or increased splicing to a downstream exon (either an unannotated distal alternative last exon or an exon in a downstream gene). Fourth, the extent of cleavage inhibition may depend on UV dose and time. Nevertheless, the main point we are making from our RNA-seq analysis is that a specific subset of genes (108 out of 4208 genes) exhibits an increase in poly(A) site cleavage efficiency upon UV irradiation.

CROSS-CONSULTATION COMMENTS
@reviewer 1 & 3: I fully agree; the PCF11 depletion under UV-conditions is clearly visible.
Thank you!

Reviewer #3 (Significance (Required)):

This is an important study to better understand the function and target genes of CoTC-mediated 3'-end processing. It thereby extends earlier studies mainly adressing the underlying molecular mechanisms and rationalizes the function (and evolution) of this gene regulatory principle.

All editorial and formatting issues were resolved by the authors.

2nd Revision - Editorial Decision 25th Jan 2023

Thank you for submitting the final revised version of your manuscript. I am happy to inform you that we have now formally accepted your study for publication in The EMBO Journal.

EMBO Press Author Checklist

Corresponding Author Name: VAGNER Stéphan	
Journal Submitted to: EMBO J	
Manuscript Number: EMBOJ-2022-112358	

USEFUL LINKS FOR COMPLETING THIS FORM
The EMBO Journal - Author Guidelines
EMBO Reports - Author Guidelines
Molecular Systems Biology - Author Guidelines
EMBO Molecular Medicine - Author Guidelines

Reporting Checklist for Life Science Articles (updated January
This checklist is adapted from Materials Design Analysis Reporting (MDAR) Checklist for Authors. MDAR establishes a minimum set of requirements in transparent reporting in the life sciences (see Statement of Task: 10.31222/osf.io/9sm4x). Please follow the journal's guidelines in preparing your
Please note that a copy of this checklist will be published alongside your article.

Abridged guidelines for figures
1. Data
The data shown in figures should satisfy the following conditions:
- the data were obtained and processed according to the field's best practice and are presented to reflect the results of the experiments in an accurate and unbiased manner.
- ideally, figure panels should include only measurements that are directly comparable to each other and obtained with the same assay.
- plots include clearly labeled error bars for independent experiments and sample sizes. Unless justified, error bars should not be shown for technical
- if n<5, the individual data points from each experiment should be plotted. Any statistical test employed should be justified.
- Source Data should be included to report the data underlying figures according to the guidelines set out in the authorship guidelines on Data

2. Captions
Each figure caption should contain the following information, for each panel where they are relevant:
- a specification of the experimental system investigated (eg cell line, species name).
- the assay(s) and method(s) used to carry out the reported observations and measurements.
- an explicit mention of the biological and chemical entity(ies) that are being measured.
- an explicit mention of the biological and chemical entity(ies) that are altered/varied/perturbed in a controlled manner.
- the exact sample size (n) for each experimental group/condition, given as a number, not a range;
- a description of the sample collection allowing the reader to understand whether the samples represent technical or biological replicates (including how many animals, litters, cultures, etc.).
- a statement of how many times the experiment shown was independently replicated in the laboratory.
- definitions of statistical methods and measures:
  - common tests, such as t-test (please specify whether paired vs. unpaired), simple χ2 tests, Wilcoxon and Mann-Whitney tests, can be unambiguously identified by name only, but more complex techniques should be described in the methods section;
  - are tests one-sided or two-sided?
  - are there adjustments for multiple comparisons?
  - exact statistical test results, e.g., P values = x but not P values < x;
  - definition of 'center values' as median or average;
  - definition of error bars as s.d. or s.e.m.

Please complete ALL of the questions below.
Select "Not Applicable" only when the requested information is not relevant for your study.

Materials

Newly Created Materials	Information included in the manuscript?	In which section is the information available? (Reagents and Tools Table, Materials and Methods, Figures, Data Availability Section)
New materials and reagents need to be available; do any restrictions apply?	Not Applicable	

Antibodies	Information included in the manuscript?	In which section is the information available? (Reagents and Tools Table, Materials and Methods, Figures, Data Availability Section)
For **antibodies** provide the following information:<br>- Commercial antibodies: RRID (if possible) or supplier name, catalogue number and or/clone number<br>- Non-commercial: RRID or citation	Yes	Materials and Methods

DNA and RNA sequences	Information included in the manuscript?	In which section is the information available? (Reagents and Tools Table, Materials and Methods, Figures, Data Availability Section)
**Short novel DNA or RNA including primers, probes**: provide the sequences.	Yes	Appendix

Cell materials	Information included in the manuscript?	In which section is the information available? (Reagents and Tools Table, Materials and Methods, Figures, Data Availability Section)
**Cell lines:** Provide species information, strain. Provide accession number in repository **OR** supplier name, catalog number, clone number, and/**OR** RRID.	Yes	Materials and Methods
**Primary cultures:** Provide species, strain, sex of origin, genetic modification status.	Not Applicable	
Report if the cell lines were recently **authenticated** (e.g., by STR profiling) and tested for mycoplasma contamination.	Not Applicable	

Experimental animals	Information included in the manuscript?	In which section is the information available? (Reagents and Tools Table, Materials and Methods, Figures, Data Availability Section)
**Laboratory animals or Model organisms:** Provide species, strain, sex, age, genetic modification status. Provide accession number in repository **OR** supplier name, catalog number, clone number, **OR** RRID.	Not Applicable	
**Animal observed in or captured from the field:** Provide species, sex, and age where possible**.**	Not Applicable	
Please detail **housing and husbandry conditions**.	Not Applicable	

Plants and microbes	Information included in the manuscript?	In which section is the information available? (Reagents and Tools Table, Materials and Methods, Figures, Data Availability Section)
**Plants:** provide species and strain, ecotype and cultivar where relevant, unique accession number if available, and source (including location for collected wild specimens).	Not Applicable	
**Microbes:** provide species and strain, unique accession number if available, and source.	Not Applicable	

Human research participants	Information included in the manuscript?	In which section is the information available? (Reagents and Tools Table, Materials and Methods, Figures, Data Availability Section)
If collected and within the bounds of privacy constraints report on age, sex and gender or ethnicity for all study participants.	Not Applicable	

Core facilities	Information included in the manuscript?	In which section is the information available? (Reagents and Tools Table, Materials and Methods, Figures, Data Availability Section)
If your work benefited from core facilities, was their service mentioned in the acknowledgments section?	Not Applicable	

Design

Study protocol	Information included in the manuscript?	In which section is the information available? (Reagents and Tools Table, Materials and Methods, Figures, Data Availability Section)
If study protocol has been **pre-registered, provide DOI in the manuscript**. For clinical trials, provide the trial registration number **OR** cite DOI.	Not Applicable	
Report the **clinical trial registration number** (at ClinicalTrials.gov or equivalent), where applicable.	Not Applicable	

Laboratory protocol	Information included in the manuscript?	In which section is the information available? (Reagents and Tools Table, Materials and Methods, Figures, Data Availability Section)
Provide DOI OR other citation details if **external detailed step-by-step protocols** are available.	Not Applicable	

Experimental study design and statistics	Information included in the manuscript?	In which section is the information available? (Reagents and Tools Table, Materials and Methods, Figures, Data Availability Section)
Include a statement about **sample size** estimate even if no statistical methods were used.	Yes	Figure Legends
Were any steps taken to minimize the effects of subjective bias when allocating animals/samples to treatment (e.g. **randomization procedure**)? If yes, have they been described?	Not Applicable	
Include a statement about **blinding** even if no blinding was done.	Not Applicable	
Describe **inclusion/exclusion criteria** if samples or animals were excluded from the analysis. Were the criteria pre-established? If sample or data points were omitted from analysis, report if this was due to attrition or intentional exclusion and provide justification.	Not Applicable	
For every figure, are **statistical tests** justified as appropriate? Do the data meet the assumptions of the tests (e.g., normal distribution)? Describe any methods used to assess it. Is there an estimate of variation within each group of data? Is the variance similar between the groups that are being statistically compared?	Yes	Figure Legends

Sample definition and in-laboratory replication	Information included in the manuscript?	In which section is the information available? (Reagents and Tools Table, Materials and Methods, Figures, Data Availability Section)
In the figure legends: state number of times the experiment was **replicated** in laboratory.	Yes	Figure Legends
In the figure legends: define whether data describe **technical or biological replicates**.	Yes	Figure Legends

Ethics

Ethics	Information included in the manuscript?	In which section is the information available? (Reagents and Tools Table, Materials and Methods, Figures, Data Availability Section)
Studies involving **human participants**: State details of **authority granting ethics approval** (IRB or equivalent committee(s), provide reference number for approval.	Not Applicable	
Studies involving **human participants**: Include a statement confirming that **informed consent** was obtained from all subjects and that the experiments conformed to the principles set out in the WMA Declaration of Helsinki and the Department of Health and Human Services Belmont Report.	Not Applicable	
Studies involving **human participants:** For publication of **patient photos**, include a statement confirming that consent to publish was obtained.	Not Applicable	
Studies involving experimental **animals**: State details of **authority granting ethics approva**l (IRB or equivalent committee(s), provide reference number for approval. Include a statement of compliance with ethical regulations.	Not Applicable	
Studies involving **specimen and field samples:** State if relevant **permits** obtained, provide details of authority approving study; if none were required, explain why.	Not Applicable	

Dual Use Research of Concern (DURC)	Information included in the manuscript?	In which section is the information available? (Reagents and Tools Table, Materials and Methods, Figures, Data Availability Section)
Could your study fall under dual use research restrictions? Please check biosecurity documents and list of **select agents and toxins** (CDC): https://www.selectagents.gov/sat/list.htm	Not Applicable	
If you used a select agent, is the security level of the lab appropriate and reported in the manuscript?	Not Applicable	
If a study is subject to dual use research of concern regulations, is the name of the **authority granting approval and reference number** for the regulatory approval provided in the manuscript?	Not Applicable	

Reporting

The MDAR framework recommends adoption of discipline-specific guidelines, established and endorsed through community initiatives. Journals have their own policy about requiring specific guidelines and recommendations to complement MDAR.

Adherence to community standards	Information included in the manuscript?	In which section is the information available? (Reagents and Tools Table, Materials and Methods, Figures, Data Availability Section)
State if relevant guidelines or checklists (e.g., **ICMJE, MIBBI, ARRIVE, PRISMA**) have been followed or provided.	Not Applicable	
For **tumor marker prognostic studies,** we recommend that you follow the **REMARK** reporting guidelines (see link list at top right). See author guidelines, under 'Reporting Guidelines'. Please confirm you have followed these guidelines.	Not Applicable	
For **phase II and III randomized controlled trials**, please refer to the **CONSORT** flow diagram (see link list at top right) and submit the CONSORT checklist (see link list at top right) with your submission. See author guidelines, under 'Reporting Guidelines'. Please confirm you have submitted this list.	Not Applicable	

Data Availability

Data availability	Information included in the manuscript?	In which section is the information available? (Reagents and Tools Table, Materials and Methods, Figures, Data Availability Section)
Have **primary datasets** been deposited according to the journal's guidelines (see 'Data Deposition' section) and the respective accession numbers provided in the Data Availability Section?	Yes	Materials and Methods/Data avialbility section
Were **human clinical and genomic datasets** deposited in a public access-controlled repository in accordance to ethical obligations to the patients and to the applicable consent agreement?	Not Applicable	
Are **computational models** that are central and integral to a study available without restrictions in a machine-readable form? Were the relevant accession numbers or links provided?	Not Applicable	
If publicly available data were reused, provide the respective **data citations in the reference list**.	Not Applicable	
